# De Novo Biosynthesis of Vindoline and Catharanthine in *Saccharomyces cerevisiae*

**DOI:** 10.34133/bdr.0002

**Published:** 2022-12-26

**Authors:** Di Gao, Tengfei Liu, Jucan Gao, Junhao Xu, Yuanwei Gou, Yingjia Pan, Dongfang Li, Cuifang Ye, Ronghui Pan, Lei Huang, Zhinan Xu, Jiazhang Lian

**Affiliations:** ^1^ Key Laboratory of Biomass Chemical Engineering of Ministry of Education, College of Chemical and Biological Engineering, Zhejiang University, Hangzhou 310027, China.; ^2^ ZJU-Hangzhou Global Scientific and Technological Innovation Center, Zhejiang University, Hangzhou 311200, China.; ^3^ Zhejiang Key Laboratory of Smart Biomaterials, Zhejiang University, Hangzhou 310027, China.

## Abstract

Vinblastine has been used clinically as one of the most potent therapeutics for the treatment of several types of cancer. However, the traditional plant extraction method suffers from unreliable supply, low abundance, and extremely high cost. Here, we use synthetic biology approach to engineer *Saccharomyces cerevisiae* for de novo biosynthesis of vindoline and catharanthine, which can be coupled chemically or biologically to vinblastine. On the basis of a platform strain with sufficient supply of precursors and cofactors for biosynthesis, we reconstituted, debottlenecked, and optimized the biosynthetic pathways for the production of vindoline and catharanthine. The vindoline biosynthetic pathway represents one of the most complicated pathways ever reconstituted in microbial cell factories. Using shake flask fermentation, our engineered yeast strains were able to produce catharanthine and vindoline at a titer of 527.1 and 305.1 μg·liter^−1^, respectively, without accumulating detectable amount of pathway intermediates. This study establishes a representative example for the production of valuable plant natural products in yeast.

## Introduction

Vinblastine, a heterodimer of catharanthine and vindoline, represents one of the most potent anticancer therapeutics [1]. Because of the complex structure with numerous stereo centers, chemical synthesis of vinblastine lacks the possibility for bulk production [2–4], while the traditional extraction method is limited by the availability of plant sources with long growth period and extremely low abundance [5,6]. In other words, current producing methods suffers from unreliable supply and high cost and cannot satisfy the growing demands. With the elucidation of vindoline and catharanthine biosynthetic pathways in recent years [7–9], the production of vinblastine using microbial cell factories holds great promise for large-scale and cost-effective production.

**Fig. 1.**
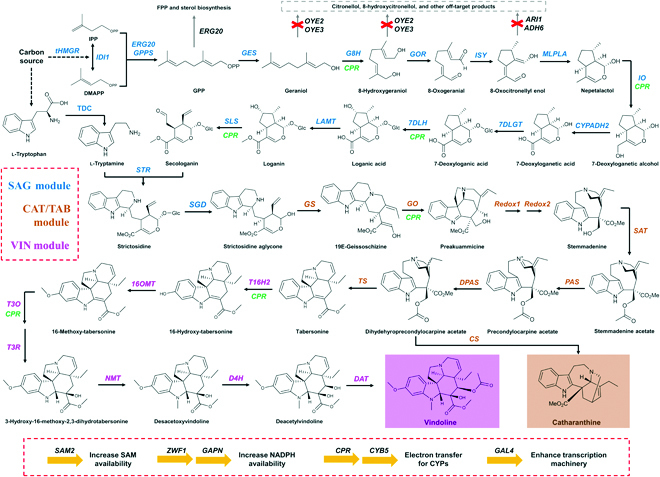
Complete biosynthesis of vindoline and catharanthine in yeast. A total of 32 heterologous genes including *CPR* and *CYB5* should be integrated to achieve de novo biosynthesis of vindoline and catharanthine. The whole biosynthetic pathway is divided into 3 functional modules: SAG module (shown in blue), from carbon sources to strictosidine aglycone; CAT/TAB module (shown in brown), from strictosidine aglycone to catharanthine/tabersonine; and VIN module (shown in magenta), from tabersonine to vindoline. The yellow arrows at the bottom represent genes overexpressed to enhance the supply of cofactors and transcriptional machinery. MLPLA, major latex protein-like gene A; SAM2, SAM synthetase; ZWF1, glucose-6-phosphate dehydrogenase; GAPN, nicotinamide adenine dinucleotide phosphate-dependent glyceraldehyde-3-phosphate dehydrogenase from *Streptococcus mutans*; CPR, CYP reductase; CYB5, cytochrome b5; GAL4, transcription activator of the yeast galactose regulon. IPP, isopentenyl pyrophosphate; DMAPP, dimethylallyl pyrophosphate; GPP, geranyl pyrophosphate; FPP, farnesyl pyrophosphate.

Recently, the entire 31 steps of vinblastine biosynthesis (Fig. 1) catalyzed by plant-specific enzymes have been elucidated in *Catharanthus roseus* [7–10]. The biosynthesis of vindoline starts from the conversion of the terpenoid pathway intermediate geranyl pyrophosphate by geraniol synthase (GES) to geraniol, followed by hydroxylation [geraniol 8-hydroxylase (G8H)], oxidation [8-hydroxygeraniol oxidoreductase (GOR)], and cyclization [iridoid synthase (ISY)] to synthesize nepetalactol. Then, nepetalactol is hydroxylated [iridoid oxidase (IO)], oxidized [alcohol dehydrogenase 2 (CYPADH2)], glycosylated [7-deoxyloganetic acid glucosyltransferase (7DLGT)], hydroxylated [7-deoxyloganic acid hydroxylase (7DLH)], and *O*-methylated [loganic acid *O*-methyltransferase (LAMT)], leading to the biosynthesis of loganin, which is subject to an unusual P450-catalyzed ring-opening reaction [secologanin synthase (SLS)]. The resultant secologanin is coupled with l-tryptamine, the decarboxylation product of l-tryptophan [tryptophan decarboxylase (TDC)], via a Pictet–Spengler-type reaction catalyzed by strictosidine synthase (STR) to form strictosidine, which is believed as the core scaffold of >3,000 monoterpenoid indole alkaloids (MIAs) [11,12]. Strictosidine is further converted to catharanthine/tabersonine by strictosidine β-d-glucosidase (SGD), geissoschizine synthase (GS), geissoschizine oxidase (GO), protein redox 1 (Redox1), protein redox 2 (Redox2), stemmadenine-*O*-acetyltransferase (SAT), precondylocarpine acetate synthase (PAS), dehydroprecondylocarpine acetate synthase (DPAS), and catharanthine synthase (CS)/tabersonine synthase (TS) [7]. Tabersonine is further hydroxylated [tabersonine 16-hydroxylase 2 (T16H2)], *O*-methylated [16-hydro-xytabersonine *O*-methyltransferase (16OMT)], hydroxylated [tabersonine 3‑oxygenase (T3O)], reduced [tabersonine 3-reductase (T3R)], *N*-methylated [3-hydroxy-16-methoxy-2,3-dihydrotabersonine-*N*-methyltransferase (NMT)], hydroxylated [desacetoxyvindoline-4-hydroxylase (D4H)], and acetylated [deacetylvindoline-4-*O*-acetyltransferase (DAT)] to generate vindoline [8,13,14]. Finally, vindoline is condensed with catharanthine to produce vinblastine by a peroxidase-mediated reaction [class III peroxidase (PRX1)] [15].

Among a series of microbial cell factories, *Saccharomyces cerevisiae* is often regarded as the top chassis for producing plant natural products including vinblastine, mainly due to advantages in the assembly of multiple genes and functional expression of cytochrome P450 enzymes (CYPs) [16,17]. Currently, several plant natural products such as artemisinic acid [18], daidzin [19], opioids [20], and scopolamine [21] have been successfully produced in *S. cerevisiae*. In terms of the vinblastine biosynthetic pathway, de novo biosynthesis of strictosidine [11] and the conversion of tabersonine to vindoline [8,13,14] have been reported in engineered yeast strains, while reconstitution of the whole biosynthetic pathway is yet to be explored. The complexity of vinblastine biosynthesis is not only represented by the longest biosynthetic pathway but also represented by the involvement of different types of enzymes (Fig. 1), such as 7 CYPs (G8H, IO, 7DLH, SLS, GO, T16H2, and T3O), 3 methyltransferases (LAMT, 16OMT, and NMT), 2 acetyltransferases (SAT and DAT), and a glucosyltransferase (7DLGT). In other words, to achieve the production of this high value compound using synthetic biology approach, challenges in manipulating multigene biosynthetic pathways, expressing rate-limiting enzymes (particularly CYPs) to high levels, and supplying sufficient amounts of various cofactors [i.e., reduced form of nicotinamide adenine dinucleotide phosphate (NADPH), *S*-adenosyl-L-methionine (SAM), acetyl-coenzyme A, and uridine diphosphate glucose] should be readily addressed.

In the present study, we aim to achieve de novo biosynthesis of vindoline and catharanthine, 2 direct precursors of vinblastine, in *S. cerevisiae*. When the manuscript is under review, Zhang et al. [22] reported a microbial supply chain for vinblastine, with the production of catharanthine and vindoline reaching as high as 91.4 and 13.2 μg·liter^−1^, respectively, in yeast using fed-batch fermentation in synthetic medium with galactose for 11 days. While the upstream strictosidine and strictosidine aglycone biosynthesis was extensively engineered, the downstream catharanthine and vindoline biosynthesis was less optimized, represented by relatively low production and accumulation of pathway intermediates (e.g., tabersonine). We, based on a previously constructed strictosidine-derivative overproducing strain [12], focus more on the construction and optimization of the catharanthine and vindoline biosynthetic pathways. Via ensuring sufficient supply of various cofactors and debottlenecking the rate-limiting steps by gene amplification, our engineered yeast strains were able to produce catharanthine and vindoline at a titer of 527.1 and 305.1 μg·liter^−1^, respectively, in 2 days using shake flask fermentation in rich medium with galactose. This study demonstrates the potential of synthetic biology for bulk production of vinblastine and other plant natural products.

## Materials and Methods

### Strains, media, and reagents

*S. cerevisiae* AJM7, derived from CEN.PK2-1C, was used as the parent strain and cultured in YPD or SED medium containing G418 sulfate (200 mg·liter^−1^; Sangon Biotech Co. Ltd., Shanghai, China). YPD medium included yeast extract (10 g·liter^−1^; Oxoid Ltd., Hants, UK), peptone A (20 g·liter^−1^; meat peptone from bovine, Sangon Biotech Co. Ltd.), and glucose (20 g·liter^−1^). The compositions of SED medium were monosodium glutamate (1.1 g·liter^−1^), yeast nitrogen base without amino acids and ammonium sulfate (1.7 g·liter^−1^ MP Biomedicals, Solon, Ohio, USA), complete supplement mixture (CSM; 0.6 g·liter^−1^; MP Biomedicals), and glucose (20 g·liter^−1^). When preparing solid medium, agar powder (20 g·liter^−1^) was added. Gene cloning and plasmid amplification were carried out in *Escherichia coli* DH5α (Tsingke Biotech, Hangzhou, China). Recombinant *E. coli* strains were selected and cultivated in LB medium containing ampicillin (100 mg·liter^−1^). Polymerase chain reaction (PCR) products were purified using the Gene JET PCR Purification Kit (Thermo Fisher Scientific, Massachusetts, USA). Plasmids were extracted from *E. coli* using the AxyPrep Plasmid Prep Kit (Axygen Biotechnology Co. Ltd., Hangzhou, China). All restriction enzymes, T4 DNA ligase, and Q5 polymerase were purchased from New England Biolabs (Beijing, China). Standard chemicals used for liquid chromatography-mass spectrometry (LC-MS) analysis were purchased from Yuanye Bio-Technology Co. Ltd. (Shanghai, China) or Chengdu Desite Biological Technology Co. Ltd. (Chengdu, China). All the other chemicals were obtained from Sigma-Aldrich (St. Louis, Missouri, USA), unless specifically indicated above.

### Plasmid construction

All plasmids were constructed by restriction/ligation, Gibson Assembly, or Golden-Gate Assembly. Genomic loci used for the integration of pathway genes and their corresponding single-guide RNA (sgRNA) plasmids were previously described [12]. The sgRNA spacer sequences were designed by Benchling (https://benchling.com) and cloned into the *Bsa*I sites of pRS423-SpSgH and/or pRS426-SpSgH. The biosynthetic pathway genes were cloned into the multiple cloning sites of pESC series vectors (pESC-HIS, pESC-TRP, pESC-LEU, and pESC-URA). Then, recombinant plasmids harboring pathway genes were used as PCR templates to obtain donor DNA fragments (pathway gene expression cassettes) with 40-base pair homology arms to the integration sites in *S. cerevisiae* genome. The catharanthine and tabersonine biosynthetic pathway genes including *GS*, *GO*, *Redox1*, *Redox2*, *SAT*, *PAS*, *DPAS*, *CS*, and *TS* from *C. roseus* were all codon-optimized for yeast and chemically synthesized by GenScript Biotech (Nanjing, China). The following recombinant plasmids harboring catharanthine and tabersonine biosynthetic pathway genes were constructed in this study, pESC-URA-*GS*-*SAT*, pESC-HIS-*PAS*, pESC-HIS-*GO*-*DPAS*, pESC-URA-*Redox2*, pESC-LEU-*Redox1*-*CS*, and pESC-LEU-*Redox1*-*TS*. The vindoline biosynthetic pathway genes including *T16H2*, *16OMT*, *T3O*, *T3R*, *NMT*, *D4H*, and *DAT* were also from *C. roseus* (gifted by V. De Luca from Brock University) and the corresponding recombinant plasmids (pESC-LEU2d-*T16H2*-*16OMT*, pESC-HIS-*NMT*-*T3R*, pESC-URA-*T3O*, and pESC-URA-*D4H*-*DAT*) were constructed in our previous studies [13]. Oligonucleotide synthesis and DNA sequencing were performed by Tsingke Biotech. Plasmids used in this study are listed in Table S1. The sgRNA plasmids and the corresponding spacer sequences are listed in Table S2. Oligonucleotides used for colony PCR verification, gene amplification, DNA sequencing, and plasmid construction are listed in Table S3. Biosynthetic pathway genes and the corresponding DNA sequences are listed in Table S4.

### Strain construction

All the modifications of the *S. cerevisiae* genome were based on the CRISPR/Cas9 system. About 1 μg of sgRNA plasmids and donor DNA fragments (pathway gene expression cassettes with 40-base pair homologous arms to the integration sites) were cotransformed into SpCas9 expressing *S. cerevisiae*-competent cells using the lithium acetate/single-stranded DNA/polyethylene glycol (LiAc/ssDNA/PEG) method [23]. Recombinant yeast strains were selected on SED-URA/G418 or SED-HIS/G418 agar plates. The desirable genome modifications (e.g., the integration of the heterologous gene expression cassettes) were initially verified by colony PCR and further confirmed by DNA sequencing. All the yeast strains constructed in this study are listed in Table, and the schematic diagram of strain construction process is shown in Fig. S1.

**Table.** Yeast strains constructed in this study.

**Strain**	**Genotype**	**Source**
AJM7	CEN.PK2-Int16::*CPR1*-*CYB5*; ΔP*_ERG20_*::P*_HXT1_*;Int9::*tGES ~ ERG20^WW^*; Int11::*G8H*; Int14::*ISY2*-*GOR*;Int4::*TDC*-*IDI1*; Int10::*GPPS2*-*tHMGR*; Int6::*G8H*;Int12::*TDC*-*CYPADH2*; Int17::*SLS2*-*STR*; Int19::*LAMT*-*7DLGT*; Int7::*IO*-*7DLH*; Int18::*SGD*-*HYS*; ΔOYE2::*ZWF1*-*GAPN*; ΔOYE3::*ERG20^WW^*-*MLPLA*; ΔARI1::*SAM2*; ΔADH6::*SLS2*-*STR*; Int5::*GAL4*	[12]
AJM7-ΔHYS	AJM7-Δ*HYS*	This study
AJM7A	AJM7-ΔHYS-IntG20::*HYS*	This study
**CAT module**		
CAT0	AJM7-ΔHYS-IntL7::*GS*-*SAT*; IntL9::*PAS*	This study
CAT2	CAT0-IntL10::*GO*-*DPAS*; IntL11::*Redox2*; IntL12::*Redox1*-*CS*	This study
CAT3	CAT2-IntL5::*GS*	This study
CAT4	CAT2-IntL5::*GO*	This study
CAT5	CAT2-IntL5::*Redox1*	This study
CAT6	CAT2-IntL5::*Redox2*	This study
CAT7	CAT2-IntL5::*SAT*	This study
CAT8	CAT2-IntL5::*PAS*	This study
CAT9	CAT2-IntL5::*DPAS*	This study
CAT10	CAT2-IntL5::*CS*	This study
**TAB module**		
TAB3	CAT0-IntL10::*GO*-*DPAS*; IntL11::*Redox2*; IntL12::*Redox1*-*TS*	This study
TAB4	TAB3-IntL5::*GS*	This study
TAB5	TAB3-IntL5::*GO*	This study
TAB6	TAB3-IntL5::*Redox1*	This study
TAB7	TAB3-IntL5::*Redox2*	This study
TAB8	TAB3-IntL5::*SAT*	This study
TAB9	TAB3-IntL5::*PAS*	This study
TAB10	TAB3-IntL5::*DPAS*	This study
TAB11A	TAB3-IntL5::*TS*	This study
**VIN module**		
VIN1	TAB4-IntG16::*16OMT*-*T16H2*; IntG17::*T3R*-*NMT*	This study
VIN3	VIN1-IntG22::*T3O*; IntG19::*D4H*-*DAT*	This study
VIN5	VIN3-IntG24::*TS*	This study
VIN7	VIN3-IntG24::*GS*	This study
VIN8	VIN3-IntG24::*DPAS*	This study
VIN10	VIN3-IntG24::*TS*; IntG25::*GS*	This study
VIN11	VIN3-IntG24::*TS*; IntG25::*DPAS*	This study
VIN12	VIN3-IntG24::*GS*; IntG15::*DPAS*	This study
VIN13	VIN3-IntG21::*TS*; IntG24::*GS*; IntG15::*DPAS*	This study

*ERG20^WW^* represents the *ERG20^F96W,N127W^* mutant.

### Fermentation

For engineered *S. cerevisiae* strains capable of de novo biosynthesis of MIAs, a single colony was inoculated into 3 ml of YPD medium and cultured for 24 h at 250 rpm and 30 °C. Then, 300 μl of seed broth was inoculated into 30 ml of YPD medium, and the culture was continued at 250 rpm and 30 °C. After 24 h, yeast cells were collected by centrifuge at 4,000 rpm for 5 min and washed twice by double-distilled H_2_O to remove residual glucose. All the yeast cells were resuspended in 30 ml of fresh YP medium containing galactose (20 g·liter^−1^) and cultured at 250 rpm and 30 °C to induce the expression of exogenous genes. Samples were taken from the fermentation broth every 24 h for qualitative and quantitative analysis. All fermentation experiments in this study were carried out with 3 biological parallels.

### Analytical methods

The fermentation broth samples were centrifuged at 12,000 rpm for 2 min, and an equal volume of ethyl acetate containing 1‰ volume of formic acid was added into the supernatant to extract the target products. Afterward, the upper organic phase was passed through a 0.22-μm membrane filter and injected for LC-MS analysis. SHIMADZU LC-MS/MS 8045 (Tokyo, Japan), equipped with an Accucore C18 high-performance LC column (100 × 2.1 mm, 2.6 μm, part no: 17126-102130, Thermo Fisher Scientific), an electrospray ionization ion source, and a triple quadrupole mass analyzer, was used for the identification and quantification of the target molecules. MIAs were monitored by using LC-MS multiple-reaction monitoring mode (MRM) with the following parameters: ajmalicine with a collision energy of 24 eV and a mass/charge ratio (*m/z*) transition from 353.15 to 144.05; catharanthine with a collision energy of 20 eV and an *m/z* transition from 337.10 to 144.15; tabersonine with a collision energy of 22 eV and an *m/z* transition from 337.40 to 305.05; vindoline with a collision energy of 28 eV and an *m/z* transition from 457.05 to 188.05; and other pathway intermediates with a positive scanned mode from *m/z* 50 to 800. The temperature of column oven was kept at 30 °C; the atomizing gas flow rate was kept at 3.0 liters·min^−1^; the pressure of collision-induced dissociation gas was maintained at 230 kPa; the desolvation line temperature was held at 250 °C; and the temperature of heating block was held at 400 °C. We used an aqueous solution of 1‰ formic acid (solvent A) and methanol (solvent B) as the mobile phases. When detecting ajmalicine and catharanthine, the gradient elution program was set as following: 90% to 10% solvent A over 25 min and returned to 90% solvent A over another 10 min with a constant flow rate of 0.3 ml·min^−1^. When detecting tabersonine and vindoline, the gradient elution program was set as follows: 90% to 5% solvent A over 40 min and returned to 90% solvent A over another 10 min with a constant flow rate of 0.3 ml·min^−1^.

## Results

### Establishment of a strictosidine aglycone platform strain

**Fig. 2.**
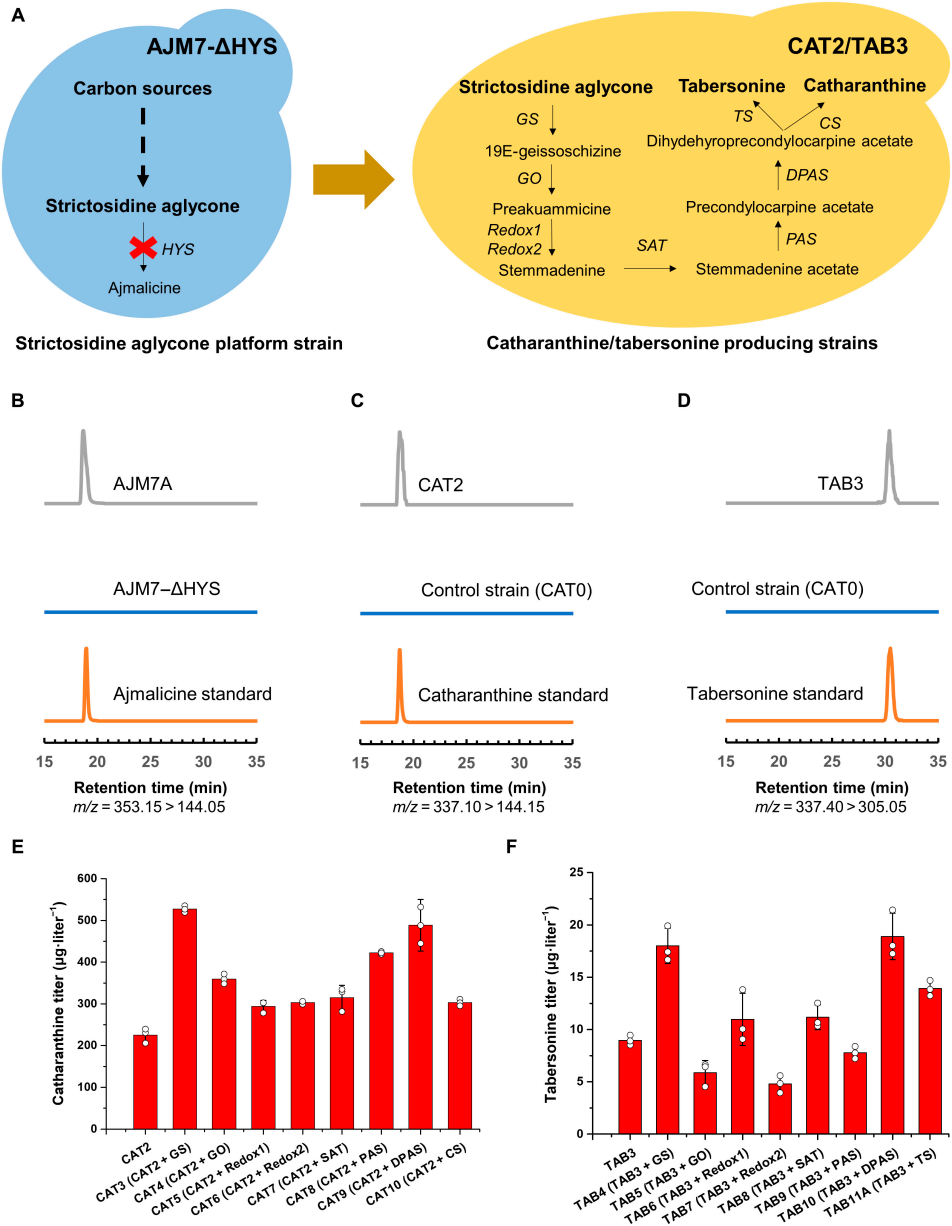
Reconstitution and optimization of CAT/TAB module for de novo biosynthesis of catharanthine and tabersonine. (A) Schematic diagram for the construction of catharanthine- and tabersonine-producing strains based on the strictosidine aglycone platform strain. (B) Construction of a strictosidine aglycone (SAG module) platform strain. LC-MS analysis of the production of ajmalicine. MRM spectra (*m/z* = 353.15 > 144.05) of ajmalicine standard, AJM7-ΔHYS (control strain) sample, and AJM7A (ajmalicine-producing strain) sample. (C) LC-MS analysis of de novo production of catharanthine in *S. cerevisiae*. MRM spectra (*m/z* = 337.10 > 144.15) of catharanthine standard, CAT0 (control strain) sample, and CAT2 (catharanthine-producing strain) sample. (D) LC-MS analysis of de novo production of tabersonine in *S. cerevisiae*. MRM spectra (*m/z* = 337.40 > 305.05) of tabersonine standard, CAT0 (control strain) sample, and TAB3 (tabersonine-producing strain) sample. (E) Metabolic pathway engineering for enhanced de novo production of catharanthine, with GS and DPAS identified as the rate-limiting enzymes. (F) Metabolic pathway engineering for enhanced de novo production of tabersonine, with GS, DPAS, and TS identified as the rate-limiting enzymes. An extra copy of each gene of the CAT/TAB module was integrated into strain CAT2 or TAB3 to enhance the production of catharanthine and tabersonine, respectively. The results represent the means ± SD of biological triplicates (*n* = 3).

As the most complicated biosynthetic pathway in nature, we divided the vindoline biosynthetic pathway into 3 functional modules, SAG module (from carbon source to strictosidine aglycone), CAT/TAB module (from strictosidine aglycone to catharanthine/tabersonine), and VIN module (from tabersonine to vindoline), with each reconstituted and optimized in a modular manner. In our previous study, we constructed a yeast strain AJM7 for overproducing ajmalicine, a strictosidine derived MIA (Fig. 2A) [12]. In addition to the introduction and optimization of the biosynthetic pathway, we removed or dynamically regulated the competing pathways (Δ*OYE2*, Δ*OYE3*, Δ*ADH6*, Δ*ARI1*, and Δ*P_ERG20_*::*P_HXT1_*) leading to the formation of nonproductive intermediates, enhanced NADPH supply via overexpression of *ZWF1* and *GAPN*, and increased SAM availability via overexpression of *SAM2*. In addition, *GAL4* was overexpressed to ensure the transcription machinery to be sufficient to drive the expression of all biosynthetic pathway genes under the control of galactose-inducible promoters (Fig. 1). Therefore, AJM7 was chosen as the parent strain for the reconstitution and optimization of the catharanthine and vindoline biosynthetic pathway. To redirect the metabolic fluxes from ajmalicine, we removed the *HYS* expression cassette using the CRISPR/Cas9 system. As shown in Fig. 2B, no ajmalicine production was detected in AJM7-ΔHYS and reintroduction of *HYS* expression cassette restored the ajmalicine-producing capability, indicating AJM7-ΔHYS as a strictosidine aglycone platform strain for reconstitution of the downstream MIA biosynthetic pathways. Notably, neither strictosidine nor strictosidine aglycone was detected in AJM7-ΔHYS, indicating that the activity of SGD was sufficient and the stability of strictosidine aglycone was too low to be accumulated. In other words, the strictosidine aglycone conversion enzyme should be active enough to channel the metabolic fluxes toward the downstream biosynthetic pathways.

### Reconstitution and optimization of CAT/TAB module for de novo biosynthesis of catharanthine and tabersonine

On the basis of the strictosidine aglycone (SAG module) platform strain, we further introduced the CAT/TAB module genes (Fig. 2A). Enabled by the previously established CRISPR/Cas9 system, we could integrate *GS*, *GO*, *Redox1*, *Redox2*, *SAT*, *PAS*, *DAPS*, and *CS*/*TS* via 2 rounds of genome editing, leading to the construction of CAT2 and TAB3. Without the supplementation of any MIA biosynthetic pathway precursors, we were able to detect the production of catharanthine (225.3 μg·liter^−1^) and tabersonine (9.0 μg·liter^−1^) in CAT2 and TAB3, respectively (Fig. 2C and D). The production of tabersonine was more than one order of magnitude lower than that of catharanthine, which was consistent with previous studies that CS was much more active than TS [22]. Although de novo biosynthesis of catharanthine and tabersonine has been achieved, their titers were still far from satisfactory and should be further improved by pathway engineering.

Because of the difficulty in quantifying pathway intermediates, we chose to identify and debottlenecked the rate-limiting steps by integrating an additional copy of each pathway gene. As shown in Fig. 2E and F, GS (CAT3) and DPAS (CAT9) were determined to be the rate-limiting enzymes of the catharanthine biosynthetic pathway, whose additional copy increased the production to 527.1 and 488.5 μg·liter^−1^, respectively; GS (TAB4), DPAS (TAB10), and TS (TAB11A) were found to be the major bottlenecks for efficient tabersonine biosynthesis and the introduction of an additional copy increased the production to 18.0, 18.9, and 14.2 μg·liter^−1^, respectively. While the pathway optimization results were almost consistent between catharanthine and tabersonine, with the last step (CS/TS) as the only exception (Fig. 2E and F). Although CS was not the bottleneck, the low activity of TS [22] made it one of rate-limiting enzymes of the tabersonine biosynthetic pathway. As the first enzyme converting the general yet unstable precursor (strictosidine aglycone) to the downstream MIA pathways, *GS* with an extra copy was beneficial for the biosynthesis of both catharanthine and tabersonine. Therefore, TAB4 with 2 copies of *GS* expression cassette integrated into the yeast chromosome was chosen for the construction of vindoline-producing strains.

### Reconstitution and optimization of VIN module for de novo biosynthesis of vindoline

**Fig. 3.**
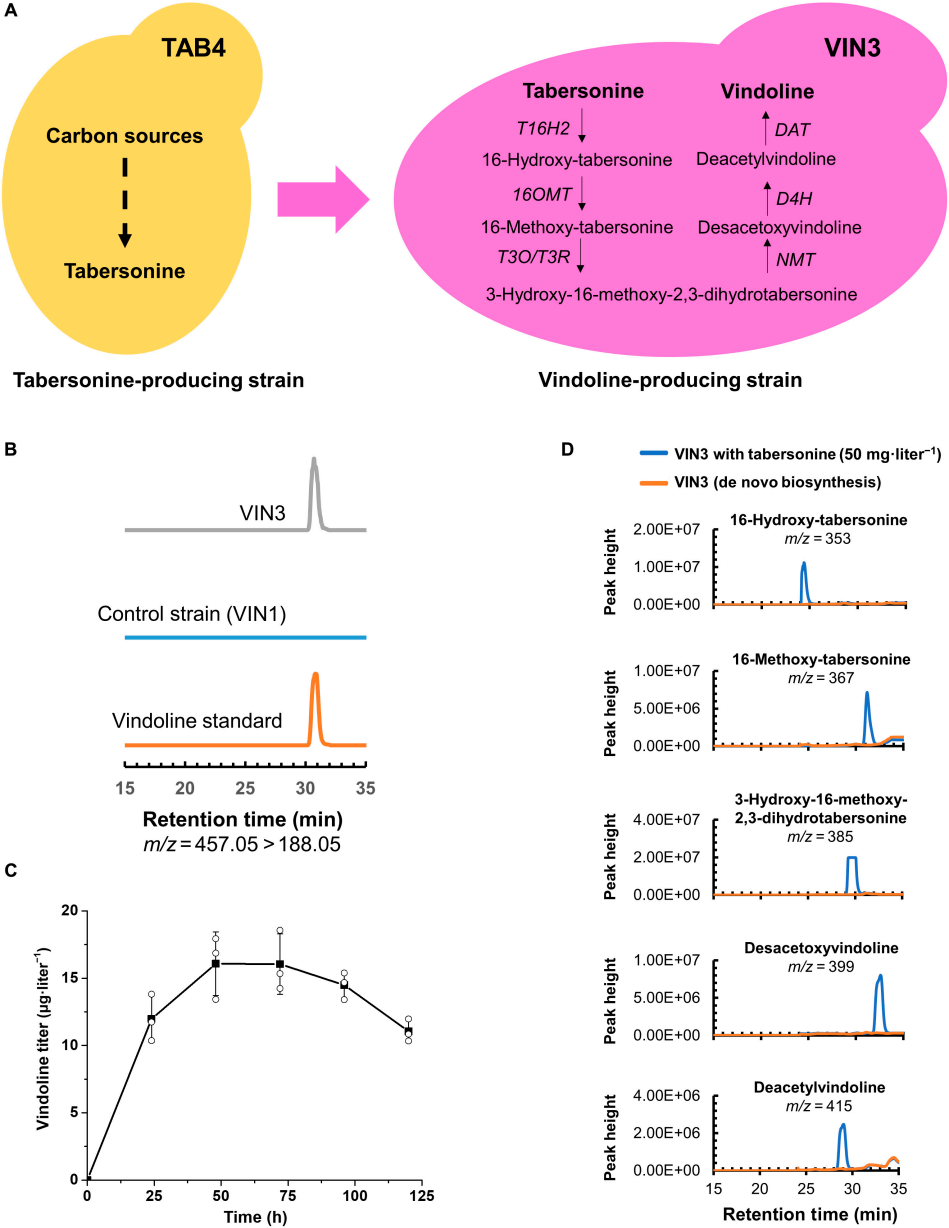
Reconstitution of VIN module for de novo biosynthesis of vindoline. (A) Schematic diagram for the construction of vindoline-producing strain based on the tabersonine-producing strain. (B) LC-MS analysis of de novo production of vindoline in *S. cerevisiae*. MRM spectra (*m/z* = 457.05 > 188.05) of vindoline standard, VIN1 (control strain) sample, and VIN3 (vindoline-producing strain) sample. (C) Time-course fermentation profile of vindoline production in VIN3. Samples were taken every 24 h after galactose induction for LC-MS analysis. (D) Effect of tabersonine concentration (with or without exogenous supplementation) on the accumulation of VIN module intermediates. Tabersonine was supplemented into the fermentation broth at a final concentration of 50 mg·liter^−1^ after galactose induction of VIN3, and the pathway intermediates were analyzed by LC-MS. 16-Hydroxy-tabersonine, 16-methoxy-tabersonine, 3-hydroxy-16-methoxy-2,3-dihydrotabersonine, desacetoxyvindoline, and deacetylvindoline were detected with *m/z* of 353, 367, 385, 399, and 415, respectively.

With an aim to achieve de novo biosynthesis of vindoline, we further introduced the VIN module genes (including *T16H2*, *16OMT*, *T3O*, *T3R*, *NMT*, *D4H*, and *DAT*) via 2 rounds of CRISPR/Cas9-based genome editing, leading to the construction of strain VIN3 (Fig. 3A). After galactose induction, we were able to detect the production of vindoline in the fermentation broth of VIN3 (Fig. 3B), representing one of the first 2 reports on the production of vindoline in a heterologous host and one of the most complicated pathways ever reconstituted in microbial cell factories. Postinduction fermentation profile revealed that the highest production (16.1 μg·liter^−1^) was achieved in 48 h (Fig. 3C), which was chosen as the time point to take samples for LC-MS analysis.

Although the vindoline production level was not sufficient for industrial applications, tabersonine was completely consumed and almost converted to vindoline. More importantly, although only one copy of each pathway gene was integrated into the yeast genome, no VIN module intermediates or by-products were accumulated to detectable levels in VIN3 (Fig. 3D). On the contrary, in our previously constructed tabersonine to vindoline conversion strains, pathway intermediates (e.g., 16-hydroxy-tabersonine and 3-hydroxy-16-methoxy-2,3-dihydrotabersonine) were accumulated to high levels and multiple copies of the pathway genes (4 copies of *T16H2* and *16OMT* as well as 2 copies of *T3O*, *T3R*, *NMT*, *D4H*, and *DAT*) should be integrated to achieve high-yield tabersonine conversion [13]. The discrepancy might result from different modes of tabersonine supply, which was gradually synthesized inside the yeast cells at a low level (<20 μg·liter^−1^) in the present study and externally supplemented at relatively high concentration (>15 mg·liter^−1^) in the previous study. To test our hypothesis, we supplemented tabersonine (50 mg·liter^−1^) after galactose induction to the fermentation broth and found several pathway intermediates (Fig. 3D) and by-products (Fig. S2) to accumulate to high levels in VIN3. These results indicated that continuous supply of tabersonine at a low concentration was beneficial for vindoline production and the VIN module with one copy of each gene integrated into the yeast genome was efficient enough to convert all tabersonine to vindoline. In other words, the availability of tabersonine was the bottleneck for de novo biosynthesis of vindoline, and the TAB module should be further engineered.

**Fig. 4.**
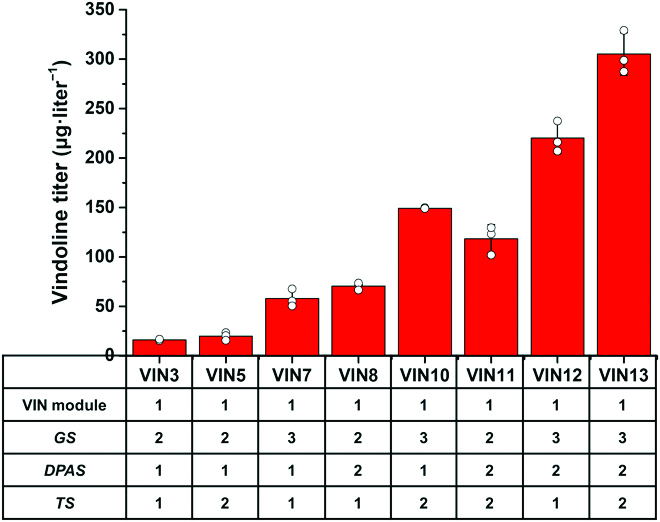
Metabolic pathway engineering for enhanced de novo production of vindoline. Additional copies of the rate-limiting enzyme encoding genes (i.e., *GS*, *DPAS*, and *TS*) were integrated into the chromosome of yeast individually and combinatorially to further increase the production of vindoline. The results represent the means ± SD of biological triplicates (*n* = 3).

To further enhance the production of vindoline in yeast, we integrated extra copies of the rate-limiting enzyme encoding genes of the TAB module (i.e., *GS*, *DPAS*, and *TS*) both individually and combinatorially. As expected, an extra copy of *GS* (VIN7), *DPAS* (VIN8), or *TS* (VIN5) expression cassette increased the production of vindoline by 3.6-, 4.4-, and 1.2-fold, respectively. Additional integration of 2 expression cassettes, *GS* and *TS* for VIN10, *DPAS* and *TS* for VIN11, and *GS* and *DPAS* for VIN12, further increased the production of vindoline to 149.3, 118.2, and 220.2 μg·liter^−1^, respectively. Nevertheless, the highest production was achieved when all 3 genes were simultaneously integrated, indicating a synergistic effect in engineering of the rate-limiting enzymes of TAB module (Fig. 4). The optimal strain VIN13 was able to produce vindoline (305.1 μg·liter^−1^) from simple carbon sources in 48 h, without the accumulation of tabersonine and VIN module intermediates to detectable levels.

## Discussion

Plant natural products play important roles in medicine, cosmetics, and food industries and are featured with complex chemical structures [16,24]. The development of synthetic biology enables scalable production of these value-added compounds in microbial cell factories, a promising alternative to the traditional plant extraction methods [25,26]. As for the biosynthesis of vinblastine precursors, although the Keasling’s group and our group both reported the complete biosynthesis of vindoline and catharanthine in *S. cerevisiae*, their production levels were still far from industrial applications. In other words, grand challenges in the manipulation of long biosynthetic pathways and the compatibility between plant enzymes and microbial cells should be readily addressed for broad applications.

In nature, vindoline biosynthesis happens in at least 5 compartments [8], with GES in the plastid, CYPs and their reductases in the endoplasmic reticulum, SGD in the nucleus, STR and PAS in the vacuole, and most of the remaining enzymes in the cytoplasm. While most of the vindoline and catharanthine biosynthetic pathway enzymes are localized in the cytoplasm of yeast, with the exception for GO, T16H2, and T3O in endoplasmic reticulum as well as PAS in vacuole, compartmentalization engineering has demonstrated advantages in minimizing competing pathways, increasing the availability of biosynthesis precursors, and providing unique environment for some specific enzymes [17,27]. For example, compartmentalization of the mevalonate pathway into mitochondrion [28,29] and/or peroxisome [30,31] dramatically increased the production of monoterpenes including geraniol, mainly due to the enhanced availability of acetyl-coenzyme A and less competition with the steroid pathway. In addition, the compartmentalization of a key enzyme (norcoclaurine synthase) into peroxisome alleviated the cytotoxicity and increased the production of a benzylisoquinoline alkaloid [32]. Therefore, compartmentalization engineering of the vindoline biosynthetic pathway will be carried out to boost production in our future studies.

Other than the division into different compartments, microbial consortia system divides multigene biosynthetic pathways into 2 or more host cells, emerging as a promising strategy to expand microbial capacity in utilizing complex and hard-to-use substrates and/or synthesizing complex products [33]. For example, an *E. coli*–*S. cerevisiae* coculture was established for de novo biosynthesis of oxygenated taxanes [34]; an *E. coli*–*Trichoderma reesei* coculture was engineered for the production of isobutanol from cellulosic biomass directly [35]. Nevertheless, the stability and tunability of population compositions are essential for optimal performance and process scale-up. Recently, Li et al. [36] established a multimetabolite cross-feeding strategy for microbial consortia design, which was demonstrated for the design and construction of a 3-strains coculture system for de novo biosynthesis of silybins. In terms of the intermediates of the vindoline biosynthetic pathway, strictosidine and tabersonine can be both exported out of the producing cells [22] and imported into the neighboring cells for downstream conversion [13], which can be employed as the branching points for the design of microbial consortia systems.

Overall, we constructed yeast strains for de novo biosynthesis of vindoline and catharanthine, representing the first 2 reports on the reconstitution of such long and complex biosynthetic pathways. In combination with cofactor supply engineering and pathway optimization strategies, our engineered yeast strains were able to produce catharanthine (527.1 μg·liter^−1^) and vindoline (305.1 μg·liter^−1^), laying a solid foundation for large-scale fermentative production of vinblastine and vincristine in near future. In addition, the strictosidine aglycone (SAG module) platform strain and CRISPR/Cas9-based modular pathway engineering strategy can be employed for bulk production of MIAs and other plant natural products.

## Data Availability

The data involved in the research are included in the manuscript and the Supplementary Materials. All relevant data are available upon reasonable request from the corresponding author.

## Supplementary Materials

Supplementary Materials Fig. S1. Strain construction procedures. Fig. S2. Effect of tabersonine concentration on the accumulation of VIN module by-products. Table S1. List of plasmids. Table S2. List of sgRNA plasmids as well as spacer sequences. Table S3. List of primers. Table S4. List of pathway gene encoding sequences.
