# Supplementary material for: De Novo Biosynthesis of Vindoline and Catharanthine in Saccharomyces cerevisiae
Source: Biodes Res. 2022 Dec 26;2022:0002. doi: 10.34133/bdr.0002 (PMC10593122; doi:10.34133/bdr.0002)
Supplement: Supplementary Materials — Fig. S1. Strain construction procedures. Fig. S2. Effect of tabersonine concentration on the accumulation of VIN module by-products. Table S1. List of plasmids. Table S2. List of sgRNA plasmids as well as spacer sequences. Table S3. List of primers. Table S4. List of pathway gene encoding sequences. [file bdr.0002.f1.docx]

**Supplementary Materials**

***De novo* Biosynthesis of Vindoline and Catharanthine in *Saccharomyces cerevisiae***

Di Gao^1^, Tengfei Liu^1^, Jucan Gao^1,2^, Junhao Xu^1,2^, Yuanwei Gou^1,2^, Yingjia Pan^1,2^, Dongfang Li^2^, Cuifang Ye^1^, Ronghui Pan^2^, Lei Huang^1,2^, Zhinan Xu^1^, Jiazhang Lian^1,2,3*^

^1^ Key Laboratory of Biomass Chemical Engineering of Ministry of Education, College of Chemical and Biological Engineering, Zhejiang University, Hangzhou 310027, China

^2^ ZJU-Hangzhou Global Scientific and Technological Innovation Center, Zhejiang University, Hangzhou 311200, China

^3^ Zhejiang Key Laboratory of Smart Biomaterials, Zhejiang University, Hangzhou 310027, China

*Corresponding author:

Prof. Jiazhang Lian (E-mail: jzlian@zju.edu.cn)

**Supplementary Figures**

**Supplementary Figure S1 Strain construction procedures.** Gene cassettes integrated in each round of genome editing and the corresponding integration sites were detailed. The production levels of catharanthine, tabersonine, and vindoline were provided as well.


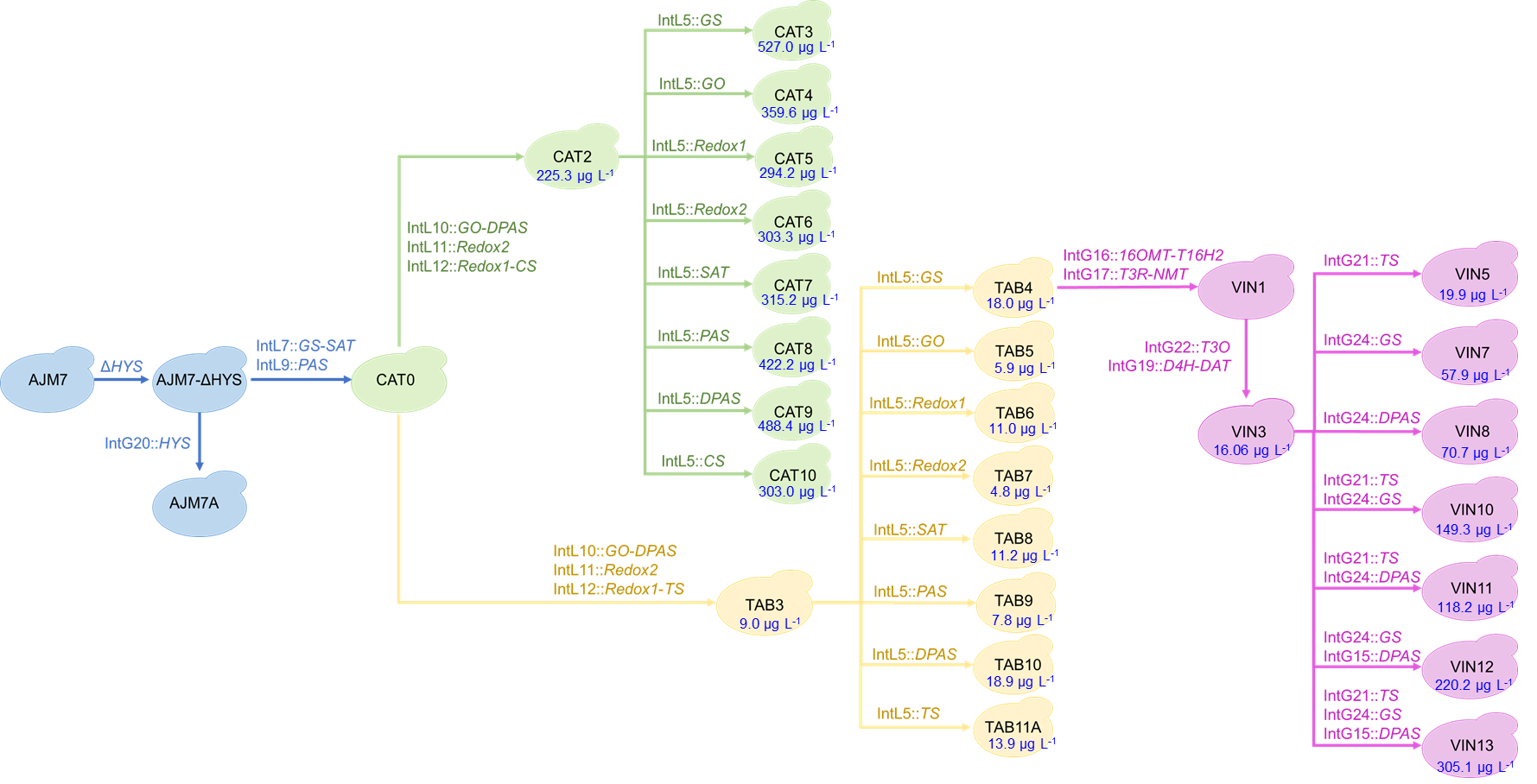


**Supplementary Figure S2 Effect of tabersonine concentration on the accumulation of VIN Module by-products.** Tabersonine was supplemented into the fermentation broth at a final concentration of 50 mg L^-1^ after galactose induction of VIN3 and the pathway by-products were analyzed by LC-MS. 3-Hydroxy-2,3-dihydrotabersonine, desacetoxyvindorosine, and vindorosine were detected with *m/z* of 355, 369, and 427.5, respectively.


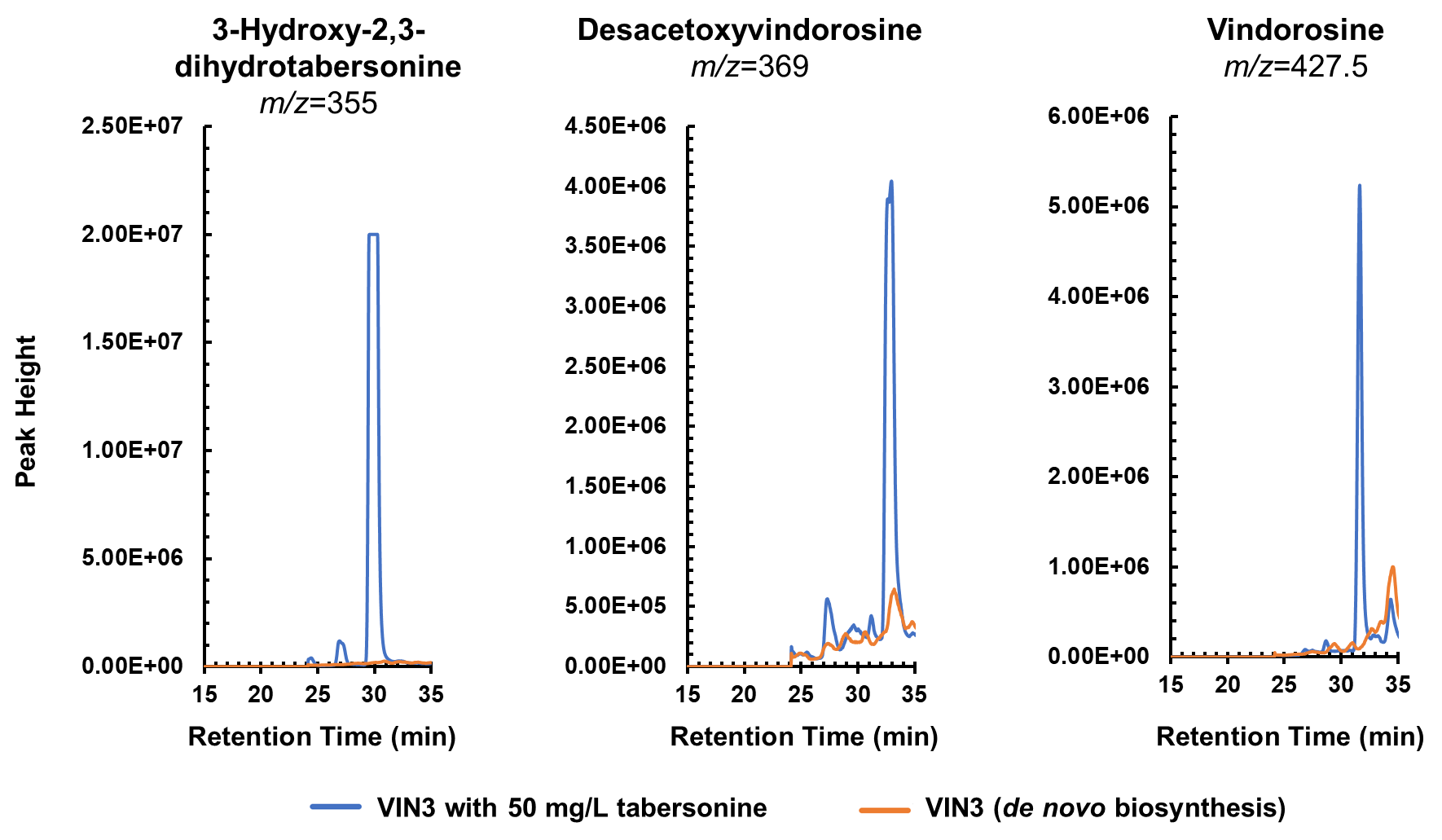


**Supplementary Tables**

**Supplementary Table S1** Plasmids used in this study

| Plasmid | Description | Source |
| --- | --- | --- |
| pESC-LEU2d-*T16H2*-*16OMT* | 2μ; *LEU2d*; *Amp*; *GAL1p*-*T16H2*-*CYC1t*; *GAL10p*-*16OMT*-*ADH1t* | [^1^](#_ENREF_1) |
| pESC-HIS-*NMT*-*T3R* | 2μ; *HIS3*; *Amp*; *GAL1p*-*NMT*-*CYC1t*; *GAL10p*-*T3R*-*ADH1t* | [^1^](#_ENREF_1) |
| pESC-URA-*T3O* | 2μ; *URA3*; *Amp*; *GAL1p*-*T3O*-*CYC1t* | [^1^](#_ENREF_1) |
| pESC-URA-*D4H*-*DAT* | 2μ; *URA3*; *Amp*; *GAL1p*-*D4H*-*CYC1t*; *GAL10p*-*DAT*-*ADH1t* | [^1^](#_ENREF_1) |
| pESC-URA-*GS*-*SAT* | 2μ; *URA3*; *Amp*; *GAL1p*-*GS*-*CYC1t*; *GAL10p*-*SAT*-*ADH1t* | This study |
| pESC-HIS-*PAS* | 2μ; *HIS3*; *Amp*; *GAL1p*-*PAS*-*CYC1t* | This study |
| pESC-HIS-*GO*-*DPAS* | 2μ; *HIS3*; *Amp*; *GAL1p*-*GO*-*CYC1t*; *GAL10p*-*DPAS*-*ADH1t* | This study |
| pESC-LEU-*Redox1*-*CS* | 2μ; *LEU2*; *Amp*; *GAL1p*-*Redox1*-*CYC1t*; *GAL10p*-*CS*-*ADH1t* | This study |
| pESC-URA-*Redox2* | 2μ; *URA3*; *Amp*; *GAL1p*-*Redox2*-*CYC1t* | This study |
| pESC-LEU-*Redox1*-*TS* | 2μ; *LEU2*; *Amp*; *GAL1p*-*Redox1*-*CYC1t*; *GAL10p*-*TS*-*ADH1t* | This study |
| pESC-LEU-*Redox1* | 2μ; *LEU2*; *Amp*; *GAL1p*-*Redox1*-*CYC1t* | This study |
| pESC-HIS-*GO* | 2μ; *HIS3*; *Amp*; *GAL1p*-*GO*-*CYC1t* | This study |
| pESC-URA-*GS* | 2μ; *URA3*; *Amp*; *GAL1p*-*GS*-*CYC1t* | This study |
| pRS423-SpSgΔ*HYS* | 2μ; *HIS3*; *Amp*; *SNR52p*-SpSgΔ*HYS*-*SUP4t* | This study |
| pESC-LEU-*SGD*-*HYS* | 2μ; *LEU2*; *Amp*; *GAL1p*-*SGD*-*CYC1t*; *GAL10p*-*HYS*-*ADH1t* | [^2^](#_ENREF_2) |

**Supplementary Table S2** List of sgRNA plasmids for genome integration of heterologous gene expression cassettes

| Plasmid | Description | Spacer sequences (5’-3’) | Chromosomal loci |
| --- | --- | --- | --- |
| pRS423-IntL5 | 2μ; *HIS3*; *Amp*; *SNR52p*-SpSgIntL5-*SUP4t* | SpSgIntL5: CACTTGTCAAACAGAATATA | ChrⅢ: 114,130-114,149 |
| pRS423-IntG19 | 2μ; *HIS3*; *Amp*; *SNR52p*-SpSgIntG19-*SUP4t* | SpSgIntG19: AGATCTTGCGAAATACTGGG | ChrⅩⅤ: 87,244-87,263 |
| pRS423-IntG20 | 2μ; *HIS3*; *Amp*; *SNR52p*-SpSgIntG20-*SUP4t* | SpSgIntG20: TAATCAGTCTAACACCCCGG | ChrⅩⅤ: 79,783-79,802 |
| pRS423-IntG21 | 2μ; *HIS3*; *Amp*; *SNR52p*-SpSgIntG21-*SUP4t* | SpSgIntG21: TCAAGGGGTTGCATATAGGG | ChrⅩⅤ: 73,653-73,672 |
| pRS423-IntG22 | 2μ; *HIS3*; *Amp*; *SNR52p*-SpSgIntG22-*SUP4t* | SpSgIntG22: TCACACGAATGAGAATTGGG | ChrⅩⅤ: 371,041-371,060 |
| pRS426-IntL11 | 2μ; *URA3*; *Amp*; *SNR52p*-SpSgIntL11-*SUP4t* | SpSgIntL11: ATATGTCTCTAATTTTGGAA | ChrⅩⅠ: 93,963-93,982 |
| pRS426-IntG16 | 2μ; *URA3*; *Amp*; *SNR52p*-SpSgIntG16-*SUP4t* | SpSgIntG16: TATATAATGAATACACATGG | ChrⅧ: 121,948-121,967 |
| pRS426-IntG21 | 2μ; *URA3*; *Amp*; *SNR52p*-SpSgIntG21-*SUP4t* | SpSgIntG21: TCAAGGGGTTGCATATAGGG | ChrⅩⅤ: 73,653-73,672 |
| pRS426-IntG22 | 2μ; *URA3*; *Amp*; *SNR52p*-SpSgIntG22-*SUP4t* | SpSgIntG22: TCACACGAATGAGAATTGGG | ChrⅩⅤ: 371,041-371,060 |
| pRS426-IntG24 | 2μ; *URA3*; *Amp*; *SNR52p*-SpSgIntG24-*SUP4t* | SpSgIntG24: AATCGGGGCAGACTATTCCG | ChrⅩⅤ: 550,742-550,761 |
| pRS426-IntG26 | 2μ; *URA3*; *Amp*; *SNR52p*-SpSgIntG26-*SUP4t* | SpSgIntG26: GAGAAAATAAAAAAAATATG | ChrⅩⅤ: 724,860-724,879 |
| pRS423-IntL10-IntL12 | 2μ; *HIS3*; *Amp*; *SNR52p*-SpSgIntL10-*SUP4t*; *SNR52p*-SpSgIntL12-*SUP4t* | SpSgIntL10: CGCCATTCAAGAGCAGCAAC SpSgIntL12: TTGTCACAGTGTCACATCAG | ChrⅩ: 236,843-236,862  ChrⅫ: 839,660-839,679 |
| pRS423-IntG16-IntG17 | 2μ; *HIS3*; *Amp*; *SNR52p*-SpSgIntG16-*SUP4t*; *SNR52p*-SpSgIntG17-*SUP4t* | SpSgIntG16: TATATAATGAATACACATGG SpSgIntG17: GAAATTATATAAAACACATG | ChrⅧ: 121,948-121,967  ChrⅧ: 147,100-147,119 |
| pRS426-IntL7-IntL9 | 2μ; *URA3*; *Amp*; *SNR52p*-SpSgIntL7-*SUP4t*; *SNR52p*-SpSgIntL9-*SUP4t* | SpSgIntL7: AATCCGAACAACAGAGCATA SpSgIntL9: GCGCCACAGTTTCAAGGGTC | ChrⅩⅥ: 776,883-776,902  ChrⅩⅣ: 280,250-280,269 |
| RS426-IntG24-IntG25 | 2μ; *URA3*; *Amp*; *SNR52p*-SpSgIntG24-*SUP4t*; *SNR52p*-SpSgIntG25-*SUP4t* | SpSgIntG24: AATCGGGGCAGACTATTCCG SpSgIntG25: TGTACTAGAAGTTCTCCTCG | ChrⅩⅤ: 550,742-550,761  ChrⅩⅤ: 113,369-113,388 |
| pRS423-IntL10-IntL11-IntL12 | 2μ; *HIS3*; *Amp*; *SNR52p*-SpSgIntL10-*SUP4t*; *SNR52p*-SpSgIntL11-*SUP4t*; *SNR52p*-SpSgIntL12-*SUP4t* | SpSgIntL10: CGCCATTCAAGAGCAGCAAC SpSgIntL11: ATATGTCTCTAATTTTGGAA SpSgIntL12: TTGTCACAGTGTCACATCAG | ChrⅩ: 236,843-236,862  ChrⅩⅠ: 93,963-93,982  ChrⅫ: 839,660-839,679 |

**Supplementary Table S3** List of oligonucleotides used in this study

| Primer | Primer sequences (5’-3’) |
| --- | --- |
| For colony PCR verification | |
| Conf-IntL5-F | ACCAACAGATATAGGCTGTGTCTTA |
| Conf-IntL5-R | TGAGCAAAACTTCCACCAGTAAACG |
| Conf-IntL7-F | GAAATGATTGCAGAAAAGCC |
| Conf-IntL7-R | CGTCATATATGAATACTAGTCAATAG |
| Conf-IntL9-F | GATTGGTTTATGATAGAATGAGCTG |
| Conf-IntL9-R | GGGTGCCTTTACCATAAAACC |
| Conf-IntL10-F | CATGGCCAATTTGGCAAATTG |
| Conf-IntL10-R | CGGTCAAAGGGGAAATCAATG |
| Conf-IntL11-F | CGTTTTTATTCAGCCATCTAACC |
| Conf-IntL11-R | GAATATACTATTTCTTCAAAATGCTTC |
| Conf-IntL12-F | GCGTCCTACAGCGTGATGA |
| Conf-IntL12-R | CCTACATCAGGACAGTAGTACC |
| Conf-IntG16-F | ATTCAAGCCTGCTGCAATTGTGAAG |
| Conf-IntG16-R | TTCAGATGACAATAGTCTCTTGCAGAACAC |
| Conf-IntG17-F | GCTAACAATGTGAATACGCACACCGTATA |
| Conf-IntG17-R | GGTTAGAAATCGCTGGTAACATTACTGATACC |
| Conf-IntG19-F | TCGCTTTATCTATCCATCAAAAGTCCGGAG |
| Conf-IntG19-R | ATTACAGTGGCGGAAAGGAGAGAG |
| Conf-IntG20-F | ACGTACTGTATCAACACAACGAAGAACGAC |
| Conf-IntG20-R | AGCAACTCCATGTAAACACCCGTACAT |
| Conf-IntG21-F | GACCAAGCTATGAAATGCGTAAGATGAAC |
| Conf-IntG21-R | ATTAAATGAGTAGATGCTGCCAGAGTACTG |
| Conf-IntG22-F | TGTAGATTCAATATATTTTCGATCAATGGCTTCTATCAG |
| Conf-IntG22-R | TCGTTCATATGTAGCAGCGATGGTAG |
| Conf-IntG24-F | TAGAGTGAATGAGCTGATGATATTTCGCCC |
| Conf-IntG24-R | TCCTGAAAGCTCGGTTTCTACTCTAGG |
| Conf-IntG25-F | AACTGTGCAACCATAACTCATGCC |
| Conf-IntG25-R | CTGTTGCTCTTCCTATATGCATTTAAATGTG |
| Conf-IntG26-F | CAGACAAACTAGGGTGAGGATTCTTCG |
| Conf-IntG26-R | TCAGTCCAATGAATAGATCGGTTAAAGC |
| For PCR amplification to obtain the donor DNA fragments | |
| IntL5-ADH1-F | AAAGATGACTAAAATAAGTGAAATTTCAACATTAACTTCGGAGCGACCTCATGCTATAC |
| IntL5-CYC1-R | TGAAACTATTGTGTAATAGAAGTGGTAGCAATATGTAGCACTTCGAGCGTCCCAAAACC |
| IntL7-ADH1-F | GAAAGAAAAACTAACACATTAATGTAGTTTTAAAATTTCAGAGCGACCTCATGCTATAC |
| IntL7-CYC1-R | TAATTTTTATTCTAGCATATATTTAAGTTTGTTTGCGAAACTTCGAGCGTCCCAAAACC |
| IntL9-ADH1-F | AGCAATAAACGTGAAATCTGTTTTGTATATATATTTGCAGGAGCGACCTCATGCTATAC |
| IntL9-CYC1-R | GTAATCTGGCATTTCATACTATCATTGCTCAAATTATCCGCTTCGAGCGTCCCAAAACC |
| IntL10-ADH1-F | CTACCAAGGTTGTTGAGGGAACACTGGGGCAATAGGCTGTGAGCGACCTCATGCTATAC |
| IntL10-CYC1-R | CTGTTACTTCTTGCAGACATCAGACATACTATTGTAATTCCTTCGAGCGTCCCAAAACC |
| IntL11-ADH1-F | TTTTAGGATATTGACGCCAAGCGTGCGTCTGATTTCTACGGAGCGACCTCATGCTATAC |
| IntL11-CYC1-R | GTTTAATAATGATCTGTATTGCTGGCTCAATCCACGTAAGCTTCGAGCGTCCCAAAACC |
| IntL12-ADH1-F | ACCGGTACCGGAGGAGACCGCTATAACCGGTTTGAATTTAGAGCGACCTCATGCTATAC |
| IntL12-CYC1-R | TTCGAATGATGAACTTGCTTGCTGTCAAACTTCTGAGTTGCTTCGAGCGTCCCAAAACC |
| IntG16-ADH1-F | TTAAGAGGGAATAAGAAAACTAAGGGAAAATACGCTATCTGAGCGACCTCATGCTATAC |
| IntG16-CYC1-R | TTACGTACTCGCATGTATTCGAAAAGCCTCTAAAAATTGCCTTCGAGCGTCCCAAAACC |
| IntG17-ADH1-F | CGAACCAGAATTTTCTTATTTTTTTCACTAAGGATAATTGGAGCGACCTCATGCTATAC |
| IntG17-CYC1-R | CAATCTTCTATTGTATTGGTGGTATTTAAGGTGAACTAATCTTCGAGCGTCCCAAAACC |
| IntG19-ADH1-F | ATCCCAACTTCTTTCTCTTCTAAACATCTATAACCTTGGAGAGCGACCTCATGCTATAC |
| IntG19-CYC1-R | AAAGTATTTCCGGGTAAGATTAAAAATGCATTCGATTTAGCTTCGAGCGTCCCAAAACC |
| IntG20-ADH1-F | GTTTTTAAACTGGTAGCAGAGCTATCCGGTAAACATGTACGAGCGACCTCATGCTATAC |
| IntG20-CYC1-R | CTTAAAGAACGCCGAGACAAGAATATGTAATGATAAGCCTCTTCGAGCGTCCCAAAACC |
| IntG21-ADH1-F | CTATAGAGGAAAAGGTTGTATATTTTAGAGATTCGTCCATGAGCGACCTCATGCTATAC |
| IntG21-CYC1-R | AGGGCAACGCTTTGAACTAGTATTTTCCGTTATAGTATGTCTTCGAGCGTCCCAAAACC |
| IntG22-ADH1-F | TTTCTATCATTGATGACGGGCATTACCCCGTTAATGACCTGAGCGACCTCATGCTATAC |
| IntG22-CYC1-R | TATTTCTCACTTATTGCACCTCTCGAAGTTCTCTCATTAGCTTCGAGCGTCCCAAAACC |
| IntG24-ADH1-F | ATGGAGTGTATAAGAATTGTAAGTTATAACACCGGCGAACGAGCGACCTCATGCTATAC |
| IntG24-CYC1-R | TATGACAATGAGGGAGAAAAGACCGCCCTTCCTTGTTCTTCTTCGAGCGTCCCAAAACC |
| IntG25-ADH1-F | GGATTCCATTTTTAATAAGGCAATAATATTAGGTATGTAGGAGCGACCTCATGCTATAC |
| IntG25-CYC1-R | TTGTGTAGAATTGCAGATTCCCTTTTACGGATTCCTAAATCTTCGAGCGTCCCAAAACC |
| IntG26-ADH1-F | ATAAGAGTGGAAAAAAAGTAACAGATTAGTGGCTCCCAGTGAGCGACCTCATGCTATAC |
| IntG26-CYC1-R | AAAATACTGTGTTGTTTGTTTCTCTCTAGCCGTTGATTGGCTTCGAGCGTCCCAAAACC |
| IntL5-GAL1-10-F | AAAGATGACTAAAATAAGTGAAATTTCAACATTAACTTCGTTTCAAAAATTCTTACTTTTTTTTTGGAT |
| IntL5-GAL1-10-R | TGAAACTATTGTGTAATAGAAGTGGTAGCAATATGTAGCAGTTTTTTCTCCTTGACGTT |
| IntG16-GAL1-10-F | TTAAGAGGGAATAAGAAAACTAAGGGAAAATACGCTATCTTTTCAAAAATTCTTACTTTTTTTTTGGAT |
| IntG16-GAL1-10-R | TTACGTACTCGCATGTATTCGAAAAGCCTCTAAAAATTGCGTTTTTTCTCCTTGACGTT |
| IntG18-GAL1-10-F | TGACCAATTAATTCTATATATTTACAAACTTTTACTGTTGTTTCAAAAATTCTTACTTTTTTTTTGGAT |
| IntG18-GAL1-10-R | TTACTTCCCAAAGACTCCTTATATTAGGATTGTCTAGACAGTTTTTTCTCCTTGACGTT |
| IntG21-GAL1-10-F | CTATAGAGGAAAAGGTTGTATATTTTAGAGATTCGTCCATTTTCAAAAATTCTTACTTTTTTTTTGGAT |
| IntG21-GAL1-10-R | AGGGCAACGCTTTGAACTAGTATTTTCCGTTATAGTATGTGTTTTTTCTCCTTGACGTT |
| IntG22-GAL1-10-F | TTTCTATCATTGATGACGGGCATTACCCCGTTAATGACCTTTTCAAAAATTCTTACTTTTTTTTTGGAT |
| IntG22-GAL1-10-R | TATTTCTCACTTATTGCACCTCTCGAAGTTCTCTCATTAGGTTTTTTCTCCTTGACGTT |
| IntG24-GAL1-10-F | ATGGAGTGTATAAGAATTGTAAGTTATAACACCGGCGAACTTTCAAAAATTCTTACTTTTTTTTTGGAT |
| IntG24-GAL1-10-R | TATGACAATGAGGGAGAAAAGACCGCCCTTCCTTGTTCTTGTTTTTTCTCCTTGACGTT |
| IntG25-GAL1-10-F | GGATTCCATTTTTAATAAGGCAATAATATTAGGTATGTAGTTTCAAAAATTCTTACTTTTTTTTTGGAT |
| IntG25-GAL1-10-R | TTGTGTAGAATTGCAGATTCCCTTTTACGGATTCCTAAATGTTTTTTCTCCTTGACGTT |
| For DNA sequencing | |
| GAL-1-10-F | TTTCAAAAATTCTTACTTTTTTTTTGGAT |
| GAL-1-10-R | GTTTTTTCTCCTTGACGTT |
| M13R-48-R | AGCGGATAACAATTTCACACAGGA |
| ADH1-F | ACAGGAAAGAGTTACTCAAGAATA |
| CYC1-R | ACTCCTTCCTTTTCGGTTAGAG |
| For plasmid construction | |
| sgRNA-DEL-HYS-F | GATCTCTAATCCAGAAGAAATGCA |
| sgRNA-DEL-HYS-R | AAACTGCATTTCTTCTGGATTAGA |
| GS-F | CCTCTATACTTTAACGTCAAGGAGAAAAAACCCCGGATCCATGGCCGGAGAAACAACCAAA |
| GS-R | GAGCGGATCTTAGCTAGCCGCGGTACCAAGCTTACTCGAGTCATTCCTCAAATTTCAATGTATTTCC |
| SAT-F | GATCTTATCGTCGTCATCCTTGTAATCCATCGATACTAGTTCAATTGCTAAAATCAGTGTCCAG |
| SAT-R | TTTGAAAATTCGAATTCAACCCTCACTAAAGGGCGGCCGCATGGCACCCCAGATGCAGATA |
| GO-F | CCTCTATACTTTAACGTCAAGGAGAAAAAACCCCGGATCCATGGAATTCTCTTTCTCCTCTCC |
| GO-R | GAGCGGATCTTAGCTAGCCGCGGTACCAAGCTTACTCGAGTTAATCATTAACCAAATGTGGAACCA |
| DPAS-F | GATCTTATCGTCGTCATCCTTGTAATCCATCGATACTAGTTTATAACTCTGACGGAGGAGTCAA |
| DPAS-R | TTTGAAAATTCGAATTCAACCCTCACTAAAGGGCGGCCGCATGGCCGGAAAATCAGCAGAA |
| Redox1-F | CCTCTATACTTTAACGTCAAGGAGAAAAAACCCCGGATCCATGGCTGATCGCGTGAAGAC |
| Redox1-R | GAGCGGATCTTAGCTAGCCGCGGTACCAAGCTTACTCGAGTCAGACAGCTACTGTTGCAT |
| Redox2-F | CCTCTATACTTTAACGTCAAGGAGAAAAAACCCCGGATCCATGGAAAAGCAAGTTGAGATC |
| Redox2-R | GAGCGGATCTTAGCTAGCCGCGGTACCAAGCTTACTCGAGTCACAAGTCTCCATCCCAAA |
| CS-F | GATCTTATCGTCGTCATCCTTGTAATCCATCGATACTAGTTTACTCATGTTTGATGAAAGATGCT |
| CS-R | TAAGAATTTTTGAAAATTCGAATTCAACCCTCACTAAAGGATGAATTCCTCAACTGATCCAACTT |
| TS-F | GCTCAGATCTTATCGTCGTCATCCTTGTAATCCATCGATATCATTTTATAAAGCTGGCG |
| TS-R | AAGAATTTTTGAAAATTCGAATTCAACCCTCACTAAAGGAATGGGGTCCTCCGATGAAA |
| PAS-F | CCTCTATACTTTAACGTCAAGGAGAAAAAACCCCGGATCCATGATAAAAAAAGTCCCAATAGTTCTTT |
| PAS-R | GAGCGGATCTTAGCTAGCCGCGGTACCAAGCTTACTCGAGTCAAAGTTCGACTTGTAAATGGAGA |

**Supplementary Table S4** List of pathway genes and the corresponding DNA sequences

| **Gene** | **Enzyme** | **Source** | **NCBI Accession No.** | **Coding sequences (5’-3’)** |
| --- | --- | --- | --- | --- |
| *HYS* | Heteroyohimbine synthase | *C. roseus* | KU865325.1 | ATGGCAGCTAAATCTCCAGAAAATGTTTACCCAGTTAAAACATTTGGTTTCGCTGCTAAAGATTCCTCCGGTTTTTTTTCCCCATTTAACTTTTCCAGAAGAGCTACCGGTGAAAATGATGTTCAATTCAAAGTTTTGTACTGTGGTACTTGTAATTACGATTTGGAAATGTCTACAAACAAGTTCGGTATGACTAAGTACCCTTTTGTTATTGGTCATGAAATTGTTGGTGTTGTTACTGAAATTGGTTCTAAGGTTCAAAAATTCAAGGTTGGTGATAAAGTTGGTGTTGGTGGTTTTGTTGGTGCTTGTGAAAAGTGTGAAATGTGTGTTAATGGTGTTGAAAATAACTGTTCTAAGGTTGAATCCACAGATGGTCATTTTGGTAATAATTTTGGTGGTTGTTGTAACATTATGGTTGTTAATGAAAAGTACGCTGTTGTTTGGCCAGAAAATTTGCCTTTGCATTCTGGTGTTCCATTGTTGTGTGCTGGTATTACTACTTATTCTCCTTTGAGAAGATACGGTTTGGATAAACCTGGTTTGAATATTGGTATTGCTGGTTTGGGTGGTTTGGGTCATTTGGCTATTAGATTTGCTAAGGCTTTTGGTGCTAAAGTTACTTTGATTTCTTCATCTGTTAAGAAGAAGAGAGAAGCTTTGGAAAAGTTTGGTGTTGATTCTTTTTTGTTGAACTCTAATCCAGAAGAAATGCAAGGTGCTTATGGTACTTTGGATGGTATTATTGATACTATGCCTGTTGCTCATTCAATTGTTCCTTTTTTGGCTTTGTTGAAGCCATTGGGTAAATTGATTATTTTGGGTGTTCCAGAAGAACCATTTGAAGTTCCTGCTCCAGCTTTGTTGATGGGTGGTAAATTAATTGCTGGTTCTGCTGCTGGTTCTATGAAAGAAACTCAAGAAATGATTGATTTCGCTGCAAAACATAATATTGTTGCTGATGTTGAAGTTATTCCTATTGATTATTTGAACACCGCTATGGAAAGAATTAAAAATTCTGATGTTAAGTACAGATTCGTTATTGATGTTGGTAATACTTTGAAGTCACCATCTTTTTAA |
| *GS* | Geissoschizine synthase | *C. roseus* | MF770507.1 | ATGGCCGGAGAAACAACCAAACTCGACCTTTCAGTGAAGGCTGTGGGATGGGGTGCTGCAGATGCATCTGGTGTCCTTCAGCCCATCAAGTTCTATAGAAGAGTCCCTGGTGAACGGGATGTGAAGATTAGAGTTTTGTACTCTGGTGTTTGCAATTTCGATATGGAAATGGTCAGAAACAAGTGGGGTTTCACCAGATATCCTTATGTGTTTGGACATGAAACTGCCGGTGAGGTGGTAGAAGTTGGCAGCAAAGTAGAGAAATTCAAGGTTGGAGACAAGGTAGCTGTGGGATGTATGGTCGGATCTTGTGGTCAATGTTATAATTGTCAAAGTGGAATGGAGAATTACTGCCCAGAGCCCAATATGGCTGATGGATCTGTTTACCGTGAGCAAGGGGAACGATCCTATGGGGGTTGTTCAAATGTGATGGTTGTTGATGAAAAGTTCGTCCTCCGATGGCCCGAAAACTTGCCTCAAGATAAAGGGGTTGCTCTCCTTTGTGCTGGGGTTGTTGTTTATAGCCCAATGAAACATTTGGGACTCGATAAGCCAGGAAAGCATATTGGGGTTTTCGGGCTGGGAGGTCTTGGTTCTGTTGCTGTTAAGTTTATTAAGGCTTTTGGTGGTAAGGCTACTGTTATTAGTACATCAAGGCGTAAGGAGAAGGAAGCCATTGAAGAACATGGTGCTGATGCTTTTGTTGTCAACACTGACTCTGAGCAATTGAAGGCTCTGGCAGGTACTATGGATGGTGTTGTGGACACCACCCCAGGTGGCCGCACTCCTATGTCACTTATGCTCAATTTGCTCAAGTTTGACGGCGCGGTTATGCTCGTAGGTGCACCGGAGTCGCTATTTGAGCTCCCTGCGGCACCTCTCATTATGGGAAGGAAAAAGATAATCGGAAGTTCCACTGGAGGTCTCAAAGAGTACCAAGAAATGCTTGATTTCGCAGCCAAACATAACATTGTATGTGATACTGAAGTTATTGGGATTGACTATCTCAGCACTGCTATGGAACGTATCAAGAATTTGGATGTCAAGTACCGTTTTGCGATTGACATTGGAAATACATTGAAATTTGAGGAATGA |
| *GO* | Geissoschizine oxidase | *C. roseus* | MF770508.1 | ATGGAATTCTCTTTCTCCTCTCCAGCCTTGTATATTGTTTATTTTTTGTTGTTCTTCGTTGTTAGACAATTGTTGAAACCAAAGTCCAAAAAGAAATTGCCACCAGGTCCAAGAACTTTGCCATTGATTGGTAATTTGCACCAATTGTCTGGTCCATTGCCACATAGAACTTTGAAGAATTTGTCTGATAAGCATGGTCCATTGATGCATGTTAAAATGGGTGAAAGAAGTGCTATTATTGTTTCTGATGCTAGAATGGCTAAGATTGTTTTGCATAATAACGGTTTGGCTGTTGCTGATAGATCTGTTAATACTGTTGCTTCAATTATGACATACAACAGTTTGGGTGTTACCTTTGCTCAATATGGTGATTATTTGACTAAGTTGAGACAAATTTACACCTTGGAATTGTTGTCTCAAAAAAAAGTTAGATCTTTTTATTCTTGTTTTGAAGATGAATTGGATACTTTTGTTAAATCTATTAAATCTAATGTTGGTCAACCAATGGTTTTGTATGAAAAAGCTAGTGCTTATTTGTATGCTACTATTTGTAGAACTATTTTTGGTTCTGTTTGTAAAGAAAAAGAAAAAATGATTAAAATTGTTAAAAAAACTTCTTTGTTGTCTGGTACTCCATTGAGATTGGAAGATTTGTTTCCATCTATGTCTATTTTTTGTAGATTTTCTAAAACTTTGAATCAATTGAGAGGTTTGTTGCAAGAAATGGATGATATTTTGGAAGAAATTATTGTTGAAAGAGAAAAAGCTAGTGAAGTTTCTAAAGAAGCTAAAGATGATGAAGATATGTTGTCTGTTTTGTTGAGACATAAATGGTATAATCCATCTGGTGCTAAATTTAGAATTACTAATGCTGATATTAAAGCTATTATTTTTGAATTGATTTTGGCTGCTACTTTGTCTGTTGCTGATGTTACTGAATGGGCTATGGTTGAAATTTTGAGAGATCCAAAATCTTTGAAAAAAGTTTATGAAGAAGTTAGAGGTATTTGTAAAGAAAAAAAAAGAGTTACTGGTTATGATGTTGAAAAAATGGAATTTATGAGATTGTGTGTTAAAGAATCTACTAGAATTCATCCAGCTGCTCCATTGTTGGTTCCAAGAGAATGTAGAGAAGATTTTGAAGTTGATGGTTATACTGTTCCAAAAGGTGCTTGGGTTATTACTAATTGTTGGGCTGTTCAAATGGACCCTACTGTTTGGCCAGAACCAGAAAAATTTGATCCAGAAAGATATATTAGAAATCCAATGGATTTTTATGGTTCTAATTTTGAATTGATTCCATTTGGTACTGGTAGAAGAGGTTGTCCAGGTATTTTGTATGGTGTTACTAATGCTGAATTTATGTTGGCTGCTATGTTTTATCATTTTGATTGGGAAATTGCTGATGGTAAAAAACCAGAAGAAATTGATTTGACTGAAGATTTTGGTGCTGGTTGTATTATGAAATATCCATTGAAATTGGTTCCACATTTGGTTAATGATTAA |
| *Redox1* | Protein redox 1 | *C. roseus* | MF770509.1 | ATGGCTGATCGCGTGAAGACCGTAGGATGGGCAGCTCACGACAGCTCCGGCTTCCTCTCTCCCTTCCAATTCACTCGAAGGGCAACAGGTGAAGAAGATGTGAGGTTGAAGGTGTTGTACTGTGGTGTCTGTCACTCAGACCTTCATAACATCAAGAACGAAATGGGATTCACCTCCTACCCTTGTGTCCCCGGGCATGAGGTTGTGGGGGAAGTGACGGAAGTGGGGAACAAAGTAAAGAAATTCATAATTGGTGATAAAGTTGGGGTTGGATTATTCGTTGACTCATGTGGCGAATGCGAACAATGTGTGAATGATGTAGAAACCTATTGTCCCAAATTGAAAATGGCTTATTTATCCATTGATGATGATGGAACTGTGATTCAAGGAGGGTACTCAAAGGAAATGGTCATCAAAGAACGCTACGTTTTCCGGTGGCCGGAAAATCTTCCTCTACCCGCCGGTACACCGCTTCTGGGTGCCGGTAGTACAGTTTATAGTCCAATGAAATACTATGGACTTGATAAGTCAGGACAACATCTAGGAGTTGTTGGCCTTGGTGGACTTGGTCATTTAGCTGTTAAATTTGCAAAGGCTTTTGGACTTAAAGTTACTGTCATTAGTACCTCTCCTAGCAAGAAGGATGAAGCCATCAATCACCTTGGTGCTGATGCATTTTTAGTTAGCACTGATCAAGAGCAAACCCAGAAAGCAATGAGCACAATGGATGGCATAATTGACACAGTATCAGCTCCTCATGCATTGATGCCATTGTTTTCCCTATTGAAACCAAATGGGAAGCTAATTGTTGTTGGTGCACCAAATAAACCAGTTGAATTAGATATTCTATTTCTTGTCATGGGAAGGAAAATGCTCGGAACATCTGCTGTTGGGGGAGTGAAGGAGACACAAGAGATGATCGATTTTGCAGCAAAACATGGTATAGTTGCAGATGTAGAAGTTGTGGAAATGGAGAATGTGAACAATGCAATGGAGCGGCTTGCGAAGGGGGATGTTAGATATAGATTTGTTCTTGATATTGGGAATGCAACAGTAGCTGTCTGA |
| *Redox2* | Protein redox 2 | *C. roseus* | MF770510.1 | ATGGAAAAGCAAGTTGAGATCCCTGAAGTAGAATTGAATTCAGGACATAAAATGCCAATTGTGGGATACGGAACATGCGTGCCAGAACCCATGCCACCGTTGGAAGAACTAACCGCAATCTTCTTAGATGCAATAAAGGTTGGTTACCGGCACTTCGACACGGCTTCGAGTTACGGCACAGAGGAGGCACTTGGTAAAGCCATAGCTGAGGCTATAAACAGCGGTTTGGTTAAAAGTAGGGAGGAATTCTTCATCAGTTGTAAGCTGTGGATTGAAGATGCAGATCATGACCTTATCTTGCCTGCCCTCAACCAGTCACTTCAGATTCTTGGGGTTGATTATTTGGATCTATATATGATACACATGCCGGTGAGAGTGAGGAAAGGTGCTCCCATGTTCAATTATTCAAAAGAGGATTTCCTTCCATTTGACATACAAGGTACATGGAAGGCCATGGAAGAGTGCAGCAAACAAGGATTGGCCAAGTCTATTGGTGTCAGCAACTACTCTGTTGAAAAACTCACTAAACTCCTAGAAACCTCCACTATTCCCCCCGCCGTTAATCAGGTTGAGATGAATGTAGCTTGGCAACAGAGAAAATTGCTGCCATTTTGCAAAGAGAAAAACATTCATATAACATCATGGTCTCCACTCCTATCTTATGGTGTCGCTTGGGGAAGTAATGCTGTCATGGAAAATCCTGTTCTCCAACAAATTGCGGCTTCCAAAGGCAAAACTGTGGCACAGGTGGCACTAAGATGGATATACGAGCAAGGAGCAAGTCTCATTACAAGGACTTCCAACAAGGACAGAATGTTTGAAAATGTTCAAATTTTTGATTGGGAACTCAGTAAAGAAGAATTGGATCAAATTCATGAAATCCCACAACGTAGGGGTACTTTAGGTGAAGAATTTATGCATCCAGAAGGACCGATCAAATCCCCAGAGGAGCTTTGGGATGGAGACTTGTGA |
| *SAT* | Stemmadenine-O-acetyltransferase | *C. roseus* | MF770511.1 | ATGGCACCCCAGATGCAGATATTGTCAGAGGAACTGATTCAACCATCATCTCCGACACCCCAAACCTTGAAAACCCATAAACTTTCCCATCTTGATCAAGTTTTATTAACATGTCATATCCCTATTATTCTCTTTTATCCAAATCAATTGGACTCAAATCTCGATCGAGCCCAAAGATCTGAGAATCTAAAACGATCTTTATCAACAGTGTTAACTCAATTTTACCCTTTAGCCGGAAGAATCAATATAAATTCTTCCGTAGATTGTAATGATTCCGGAGTCCCTTTTCTTGAAGCTCGAGTTCATTCCCAACTCTCAGAAGCAATTAAAAATGTCGCCATAGATGAACTCAATCAATACCTGCCATTCCAACCTTATCCCGGTGGGGAGGAAAGTGGGTTGAAAAAAGATATCCCCTTAGCTGTAAAAATCAGTTGTTTCGAATGTGGCGGAACAGCAATTGGGGTCTGTATTTCCCACAAGATTGCCGATGCGTTGTCCTTGGCTACCTTCCTCAATTCATGGACCGCAACATGCCAGGAAGAGACTGATATTGTTCAACCTAATTTCGATCTGGGATCCCATCATTTTCCGCCTATGGAAAGCATCCCAGCACCTGAATTTCTACCGGATGAAAATATTGTGATGAAACGGTTCGTGTTCGATAAAGAAAAATTAGAAGCTCTAAAAGCCCAATTAGCTTCCTCTGCAACAGAAGTGAAGAATTCAAGTCGGGTACAAATTGTTATTGCTGTTATATGGAAGCAATTCATTGACGTGACCCGGGCAAAATTCGATACCAAGAACAAATTAGTAGCAGCTCAAGCAGTGAATTTGAGATCAAGAATGAATCCACCATTTCCTCAATCTGCTATGGGGAATATAGCCACAATGGCATATGCCGTTGCAGAGGAGGATAAGGATTTTTCAGATCTTGTCGGTCCATTGAAAACCAGTCTTGCAAAAATTGATGATGAACATGTTAAAGAATTACAAAAAGGAGTAACATATTTGGATTATGAAGCTGAACCACAAGAATTGTTCTCTTTTAGTAGTTGGTGCAGGCTTGGGTTTTATGATTTGGATTTTGGATGGGGAAAGCCTGTTTCTGTTTGTACAACAACTGTGCCTATGAAGAATTTGGTATATTTGATGGATACAAGAAATGAAGATGGAATGGAAGCATGGATCAGCATGGCTGAAGATGAGATGTCTATGCTTTCCTCTGATTTTCTTTCACTTCTGGACACTGATTTTAGCAATTGA |
| *PAS* | Precondylocarpine acetate synthase | *C. roseus* | MH136588.1 | ATGATAAAAAAAGTCCCAATAGTTCTTTCAATTTTCTGCTTTCTTCTTCTACTCTCATCATCCCATGGCTCAATTCCTGAAGCTTTTCTCAATTGTATTTCCAATAAATTTTCATTAGATGTATCCATTTTAAACATTCTTCATGTTCCCAGCAATTCTTCCTATGATTCTGTTCTCAAATCTACTATCCAAAATCCAAGATTCCTCAAATCACCCAAGCCCTTAGCTATAATCACCCCAGTACTTCACTCCCATGTCCAATCTGCTGTTATCTGTACCAAACAAGCCGGTTTACAAATTAGAATCCGAAGCGGAGGAGCTGATTACGAGGGCTTATCCTATCGTTCTGAGGTTCCCTTTATTCTGCTAGATCTCCAGAATCTTCGATCAATTTCCGTTGATATTGAAGACAACAGCGCTTGGGTCGAATCAGGAGCAACAATTGGTGAATTCTATCATGAGATAGCTCAGAACAGCCCTGTTCATGCGTTTCCAGCTGGGGTCTCTTCCTCTGTTGGAATTGGCGGCCATTTGAGTAGCGGCGGTTTTGGTACATTGCTTCGGAAATATGGATTAGCAGCCGATAATATAATCGATGCAAAAATTGTTGATGCCAGAGGCAGAATTCTTGATAGGGAATCAATGGGAGAAGATCTATTTTGGGCTATTAGAGGAGGAGGAGGAGCTAGTTTTGGTGTTATAGTTTCTTGGAAGGTTAAACTTGTAAAAGTCCCTCCGATGGTAACTGTTTTCATCTTGTCCAAGACTTATGAAGAAGGAGGTTTAGATCTTCTACACAAATGGCAATATATAGAACACAAACTCCCTGAAGATTTATTCCTTGCTGTAAGCATCATGGATGATTCATCTAGTGGAAATAAAACACTTATGGCAGGTTTTATGTCTCTGTTTCTTGGAAAAACAGAGGACCTTCTGAAAGTAATGGCGGAAAATTTCCCACAACTTGGATTGAAAAAGGAAGATTGCTTAGAAATGAATTGGATTGATGCAGCAATGTATTTTTCAGGACACCCAATTGGAGAATCCCGATCTGTGCTTAAAAACCGAGAATCTCATCTTCCAAAGACATGCGTTTCGATCAAATCAGACTTTATTCAAGAACCACAATCCATGGATGCATTGGAAAAGTTATGGAAGTTTTGTAGGGAAGAAGAAAATAGTCCCATAATACTGATGCTTCCACTGGGGGGAATGATGAGTAAAATATCAGAATCAGAAATCCCATTTCCTTACAGAAAAGATGTGATTTACAGTATGATATACGAAATAGTTTGGAATTGTGAAGACGATGAATCATCGGAAGAATATATCGATGGATTGGGAAGGCTTGAGGAATTAATGACTCCATATGTGAAACAACCAAGAGGTTCTTGGTTCAGCACCAGAAACCTTTATACCGGTAAAAATAAAGGTCCAGGAACAACTTATTCCAAAGCTAAAGAATGGGGATTTCGGTATTTTAATAATAATTTCAAAAAGTTGGCCCTTATCAAAGGACAAGTTGATCCAGAAAACTTCTTCTACTATGAACAAAGCATTCCCCCTCTCCATTTACAAGTCGAACTTTGA |
| *DPAS* | Dehydroprecondylocarpine acetate synthase | *C. roseus* | KU865331.1 | ATGGCCGGAAAATCAGCAGAAGAAGAACATCCCATTAAGGCTTACGGATGGGCTGTTAAAGATAGAACAACTGGGATTCTTTCTCCCTTCAAATTTTCCAGAAGGGCAACAGGTGATGATGATGTCCGAATTAAGATACTCTACTGTGGAATTTGTCACACTGATCTTGCCTCAATCAAGAACGAATACGAGTTTCTTTCTTATCCTCTTGTGCCCGGGATGGAGATCGTTGGAATAGCAACGGAGGTTGGAAAAGATGTCACAAAAGTGAAAGTTGGCGAAAAAGTAGCATTATCAGCCTATTTAGGATGTTGTGGCAAATGCTATAGTTGTGTAAATGAACTCGAGAATTATTGTCCGGAAGTAATCATAGGTTATGGCACCCCATACCATGACGGAACAATTTGCTATGGGGGCCTTTCAAACGAAACTGTCGCAAATCAAAGTTTTGTTCTTCGTTTTCCTGAAAGACTTTCTCCAGCTGGCGGAGCTCCTTTGCTTAGCGCCGGAATTACTTCGTTTAGTGCAATGAGAAATAGCGGCATCGACAAACCTGGATTACACGTGGGAGTCGTCGGTCTCGGCGGATTAGGTCATCTTGCTGTAAAATTTGCTAAGGCTTTTGGTCTTAAAGTAACTGTTATTAGCACCACTCCCAGCAAGAAGGATGATGCTATAAATGGTCTTGGTGCTGATGGATTCTTACTCAGCCGCGATGATGAACAAATGAAGGCTGCTATTGGAACCTTGGATGCAATTATTGATACACTGGCGGTTGTTCATCCCATAGCACCATTGCTTGATCTCCTGAGAAGTCAAGGGAAATTTTTGTTACTTGGGGCGCCATCTCAATCACTTGAGTTGCCACCTATTCCTTTATTATCAGGTGGGAAATCTATCATTGGAAGTGCGGCCGGAAATGTGAAGCAAACTCAAGAAATGCTTGATTTTGCAGCGGAGCATGATATAACTGCAAATGTTGAGATTATTCCAATAGAGTACATAAATACTGCAATGGAACGTTTAGACAAGGGCGATGTTAGATACCGATTTGTAGTTGACATCGAAAATACCTTGACTCCTCCGTCAGAGTTATAA |
| *CS* | Catharanthine synthase | *C. roseus* | MF770512.1 | ATGAATTCCTCAACTGATCCAACTTCAGATGAGACTATTTGGGATCTTTCTCCATATATTAAAATTTTCAAAGATGGAAGAGTAGAAAGACTCCATAATAGTCCTTATGTTCCCCCATCACTTAATGATCCAGAAACTGGCGTTTCTTGGAAAGATGTCCCGATTTCATCACAAGTTTCCGCTAGGGTATACATTCCAAAAATCAGCGACCATGAAAAACTCCCTATTTTTGTGTATGTGCATGGGGCTGGCTTTTGTCTAGAATCTGCCTTCAGATCATTTTTCCACACTTTTGTCAAACACTTCGTAGCCGAAACCAAAGTTATTGGGGTTTCGATTGAATATAGACTTGCCCCAGAGCACCTTTTACCCGCAGCTTATGAAGATTGTTGGGAAGCCCTTCAATGGGTTGCTTCTCATGTGGGTCTCGACAATTCCGGCCTAAAGACAGCTATTGATAAAGATCCATGGATAATAAACTATGGTGATTTCGATAGACTGTATTTGGCGGGTGACAGTCCTGGTGCTAATATTGTTCACAACACACTTATCAGAGCTGGAAAAGAGAAACTGAAGGGCGGAGTGAAAATTTTGGGGGCAATTCTTTACTACCCATATTTCATTATCCCAACCAGCACGAAACTTAGTGATGATTTTGAGTATAACTACACATGTTACTGGAAATTGGCTTATCCAAATGCTCCTGGCGGGATGAATAACCCAATGATAAACCCCATAGCTGAAAATGCTCCAGACTTGGCTGGATACGGTTGCTCGAGGTTGTTGGTTACCCTGGTTTCCATGATTTCAACGACTCCAGATGAGACTAAAGACATAAATGCGGTTTATATTGAGGCATTAGAAAAGAGTGGATGGAAAGGGGAATTGGAAGTGGCTGATTTTGACGCAGATTATTTTGAACTCTTCACCTTGGAAACGGAGATGGGCAAGAATATGTTCAGACGTTTAGCATCTTTCATCAAACATGAGTAA |
| *TS* | Tabersonine synthase | *C. roseus* | MF770513.1 | ATGGGGTCCTCCGATGAAACCATTTTTGACCTTCCTCCTTACATAAAGGTATTCAAGGATGGCCGCGTCGAAAGACTGCACTCAAGTCCCTACGTACCACCAAGCTTAAACGACCCAGAGACTGGCGGAGTCTCATGGAAGGACGTTCCCATCAGCTCTGTGGTTTCGGCAAGGATATATCTACCGAAAATCAACAACCACGATGAAAAGCTTCCAATTATTGTCTATTTTCATGGTGCGGGCTTCTGCTTGGAGTCTGCCTTCAAATCATTCTTCCACACCTATGTGAAACATTTTGTAGCGGAGGCCAAAGCAATTGCCGTTTCCGTAGAATTCCGTCTTGCTCCTGAGAATCATCTACCAGCTGCTTACGAGGATTGTTGGGAAGCACTTCAATGGGTCGCTTCGCACGTGGGACTAGACATCTCATCGCTGAAAACGTGCATTGATAAGGACCCGTGGATTATTAATTATGCGGACTTTGACCGACTCTACCTCTGGGGGGACTCGACAGGGGCCAATATCGTTCATAACACTTTGATCAGATCAGGCAAGGAAAAACTCAATGGAGGGAAAGTGAAGATACTGGGTGCTATATTATACTATCCGTATTTCTTGATCCGGACGTCCAGTAAACAGAGCGACTACATGGAGAATGAGTATCGAAGTTACTGGAAATTAGCGTATCCTGACGCACCGGGTGGTAATGATAACCCCATGATAAACCCCACAGCCGAGAACGCCCCCGATCTAGCAGGATACGGATGTAGTCGGCTACTTATCTCTATGGTAGCGGACGAGGCTAGGGATATAACATTATTGTATATAGACGCACTGGAGAAGTCTGGCTGGAAGGGTGAACTGGATGTGGCGGATTTTGATAAACAGTACTTCGAGTTATTTGAAATGGAAACGGAAGTCGCTAAGAATATGTTGCGCCGTCTCGCCAGCTTTATAAAATGA |
| *T16H2* | Tabersonine 16-hydroxylase | *C. roseus* | JF742645.1 | ATGGAGTTGTATTATTTTTCCACCTTTGCCTTCCTTCTTTTCTGCTTCATTTTAGCCAAAACTCTAAAGAAATCTGGCCAATCAAATCTTAAGCTGCCTCTGGGGCCTCCCCCAATTCCTATATTAGGAAATGCCCATCAACTTATAGGTGGCCATACTCATCACATTCTAAGAGATTTGGCCAAAAAATATGGACCGTTGATGCACTTAAAGACTGGTGAAGTTTCAACCATTGTTGCATCCTCACCAGAAATTGCTGAAGAGATGTTTAAAACACATGATGTTCTCTTTGCCGACAGACCCTCAAATATTGTTGCCTTCAAAATCTTGTCTTATGATTATTCGGATGTTGTCATTAGTCCATATGGCAATTATTGGAGACAACTTCGTAAAATTAGCATGATGGAGCTTTTTAGCCAAAGGAGTGTTCAATCTTTTAGATCAATTAGAGAAGAGGAAGTATTGAATTTTATTAAATCAATTGGTTCGAGAGAGGGTACAAAAATTAATCTTAGCAAGGAAATATCGTTACTTATTTATGGAATTACTACGCGTGCTGCTTTTGGAGAAAAAAATAAGAATACAGAAGAATTTATTCGTCTTCTTGATCAACTCACAGTGGCAGTCGCGGAACCTAACATTGCAGATATGTTCCCCTCTATCAATTTTCTTAAATTAATTAGTAGATCGAAATATAAGATTGAGAAAATACACAAAAATTTTGATGCCATAGTTCAAACTATTCTCAACCATCATAAGGATAGATTAGCCAATCACAAGTCCTCAAGTCATGAAGAGAATGGGGAACAAAACAAGGATCTTGTTGATGTTCTACTCAATATTCAACAACGTGGTGATTTTGATACACCACTAGGTGATCGCAGCGTCAAAGCAGTAATTTTTAACATATTCAGTGCCGGAACTGAGACATCGTCAACGACAGTGGATTGGGCCATGTGTGAAATGATAAAAAATCCAACGATAATGAAAAAGGCACAAGAAGAGGTAAGAAAGGTATATAATGAAGAAGGAAATGTTAATGAAACAAAACTTCATCAGCTAAAATATTTAAAAGCAGTGATTAAAGAAACATTAAGGCTTCATCCACCAGTTCCATTACTACTTCCAAGAGAATGTCGAGAACAATGTGAGATTAAAGGGTACACAATACCATCCAAATCTAGAGTTATAGTCAATGCATGGGCTATCGGAAGGGATCCGAATTACTGGATTGAACCTGAAAATTTTAACCCGGAGAGATTTCTTGAATCAGAAGTTGATTTTAAAGGAAACTCATTCGAGTATCTGCCGTTTGGTGGTGGAAGAAGGATATGTCCGGGCATAACATTTGCTTTGGCTAATATAGAACTGCCATTAGCACAACTTTTGTTCCATTTTGATTGGAAACTTGCCAGTGATGAAACAAATATTGATAAATTAGACATGACGGAGAGTAGAGGGGTAACAGTTAGAAGAGAAGATGATTTGTGTCTGATTCCATTTCCTTATTCTGCTTCTTCTCTCAAAGGTAAATATTAG |
| *16OMT* | 16-Hydroxytabersonine O-methyltransferase | *C. roseus* | EF444544.1 | ATGGATGTTCAATCTGAGGAGTTCCGTGGAGCACAAGCTCAAATATGGAGCCAATCCTGCTCTTTCATAACTTCTGCTTCTCTAAAATGCGCAGTTAAATTAGGAATTCCAGATACAATTGACAATCATGGAAAACCCATTACTCTTTCTGAATTAACCAATGCTCTTGTGCCCCCAGTTCACCCTTCTAAAGCTCCTTTTATCTACCGCCTAATGCGTGTTTTAGCAAAAAATGGCTTCTGTTCTGAAGAACAATTAGATGGAGAAACAGAACCCCTTTATTCCCTTACCCCATCAAGTCGAATTTTATTAAAAAAAGAACCCTTAAATTTAAGAGGAATTGTTCTTACCATGGCTGACCCTGTTCAACTTAAAGCTTGGGAATCCTTAAGTGATTGGTATCAAAATGAAGACGATTCATCAACTGCTTTTGAAACAGCTCATGGAAAGAATTTCTGGGGTTATAGTTCTGAACATATGGAACATGCTGAATTTTTCAATGAAGCTATGGCTAGTGATTCTCAATTGATTTCAAAGTTGTTGATTGGAGAGTATAAGTTCTTGTTTGAGGGCTTGGCTTCTCTTGTTGATATAGGAGGCGGTACCGGTACAATTGCTAAAGCCATTGCCAAGAATTTTCCTCAATTAAAGTGTACTGTGTTTGATCTTCCACATGTTGTTGCTAATCTTGAATCAAAGGAGAATGTAGAATTTGTTGCAGGTGACATGTTTGAGAAAATACCCTCTGCTAATGCTATCTTTCTCAAGTGGATTCTCCATGACTGGAACGATGAAGATTGTGTGAAGATACTCAAAAGCTGCAAAAAAGCAATTCCAGCGAAGGGTGGAAAGGTGATAATCATAGACATGGTAATGTATAGCGACAAAAAAGATGACCATTTGGTTAAAACACAGACATCTATGGATATGGCAATGCTTGTAAATTTTGCTGCTAAAGAAAGATGTGAGAAAGAATGGGCATTTCTCTTTAAGGAAGCTGGTTTTAGTGATTACAAGATTTATCCTAAATTGGATTTTACAAGGAGTCTCATTGAGGTTTATCCTTGA |
| *T3O* | Tabersonine 3-oxygenase | *C. roseus* | I1TEM1.1 | ATGGAGTTTCATGAATCTTCTCCCTTCGTCTTCATCACTCGTGGCTTTATATTCATAGCAATTTCAATAGCCGTACTGAGAAGAATAATATCAAAGAAAACTAAAACATTGCCTCCAGGACCATGGAAGCTACCTTTGATTGGAAATCTTCATCAATTTCTGGGTTCTGTTCCTTATCAAATTCTCAGAGATTTAGCTCAAAAAAATGGGCCTTTGATGCACCTTCAATTAGGTGAAGTTTCTGCCATCGTGGCAGCATCTCCTCAAATGGCTAAGGAGATTACAAAAACTTTGGATCTTCAATTTGCAGACAGGCCAGTTATTCAAGCATTAAGGATTGTGACCTATGATTATTTAGATATATCCTTTAATGCATACGGAAAATATTGGAGACAATTGCGTAAAATTTTTGTCCAAGAACTATTAACTTCAAAGAGAGTTCGATCATTTTGCTCTATAAGAGAAGATGAATTTTCCAATCTGGTAAAAACAATCAATTCTGCGAATGGAAAATCAATCAATTTGAGCAAATTGATGACGTCATGCACAAATTCAATTATTAATAAAGTAGCTTTTGGTAAAGTACGTTATGAACGGGAGGTGTTTATTGATCTAATTAATCAAATATTAGCATTAGCAGGCGGTTTTAAGCTGGTTGATCTGTTTCCGTCCTACAAGATACTTCATGTTCTTGAAGGTACAGAACGTAAGCTGTGGGAAATCCGCGGTAAGATTGACAAGATTTTGGATAAAGTCATAGACGAGCACAGAGAAAATTCGTCAAGAACTGGAAAGGGCAACGGTTGTAATGGCCAGGAAGATATAGTTGATATTTTACTTAGGATTGAAGAGGGTGGTGATCTTGACCTTGATATTCCCTTTGGCAACAACAATATCAAAGCTCTTTTATTCGATATAATTGCAGGTGGAACTGAAACCTCATCAACAGCAGTTGACTGGGCAATGTCAGAGATGATGAGAAATCCCCATGTGATGAGCAAAGCGCAAAAGGAAATTAGGGAAGCGTTCAATGGAAAGGAGAAGATTGAGGAGAATGATATTCAAAATTTGAAGTACCTAAAGTTAGTGATCCAAGAAACCTTAAGGTTACACCCTCCTGCTCCATTGTTGATGAGACAATGCCGAGAGAAATGTGAAATTGGCGGATATCATATACCTGTTGGAACAAAAGCGTTCATCAATGTCTGGGCAATCGGAAGGGATCCTGCGTATTGGCCTAATCCAGAGAGTTTTATTCCGGAAAGATTTGACGATAATACTTATGAATTTACAAAATCTGAACATCATGCGTTTGAATATTTGCCATTTGGTGCCGGAAGAAGGATGTGTCCGGGCATTTCATTTGGTTTGGCCAACGTGGAGCTTCCTTTAGCTCTACTTCTTTACCATTTCAACTGGCAACTCCCAGATGGTTCTACTACTCTGGATATGACAGAGGCTACTGGATTAGCAGCAAGAAGAAAATATGATCTTCAATTAATCGCTACGTCCTATGCATGA |
| *T3R* | Tabersonine 3-reductase | *C. roseus* | KP122966.1 | ATGGCTGCAAAGTCAGTGAAGGCTCTTGGTTTGGCTCTTAAGGATTCATCTGGGCTTTTCTCTCCATTCAACTTCTCAAGAAGGGCTACAGGGGAACACGATGTTCAATTGAAAGTATTATATTGTGGTGTTTGCAATTTCGATAACTTAATGAGGAGAAACAAATACGGGAGGACCAAGTTTCCCTATGTTTTCGGGCATGAAATTGTGGGTGTTGTAACTGAGGTTGGTTCCAATGTTAAGAAATTCAAAATCGGTAACAAAGTTGGGGTTAGCTTTATCGTTGATACCTGTAGAGAATGTGAAAGGTGTAAAATTGGACAGCAAATAGCCTGTAAAAAAGCAGTATCATCGGACGGCTTCTTCGAGACACCAGGTTACGGTGGTTGTTCAAATATATTTGTGGCTGACGAGAATTATGTCATACTTTGGCCTGAAAACCTTCCTATGGATTCTGGGGCACCCCTGCTGTGTATAGGAATTACATGTTATAATCCCTTAAGACGTTTTGGACTTGATAAACCTGGAGTTAGAGTTGGTATAGTTGGTCTAGGAGCAGTTGGACATTTAGCTATTAAATTTGCAAAAGCTTTTGGTGCTAGGGTTACTTTGATCAGTTCATCCCCTGGAAAAAAGGATGAGGCTTTTCAGAAATTTGGTGTAGATTCTTTCTTGGTCAGCAGTAATGCAGAGGAAATGCAGGCTGCAGCTGAAACTCTGGATGGTATCCTAGACACTGTACCAGTGGTTCACCCCCTTGAGCCACTCTTTGCTTTACTGAAACCTCTTGGGAAACTTATCATTATAGGTGAACCGCATAAGCCTTTTGAGGTATCCGCAATGTCCCTCATGGAGGGTGGAAAAATAATTAGCGCGAGTACGGGTGGAAGTATAAAGGACACACAAGAGATAGTCGATTTTGCAGCAGAACATAACGTAGTTGCAGATGTTGAGGTTATCCCCGTGGACTATGTGAACACTGCCATGGAGCGTCTTGATAAAGCTGATGTGAAGTATCGTTTCGTGATTGACATTGGAAACACTTTCAAATCACCCTAA |
| *NMT* | 3-Hydroxy-16-methoxy-2,3-dihydrotabersonine-N-methyltransferase | *C. roseus* | HM584929.1 | ATGGAAGAGAAGCAGGAGAAGGTGGCTGAGTTCTATGACAAAGTAACCGGAGCATGGGATCTCTTCTATGGAGTCCATTTACATGATGGATATTATGAGCCTGGAACTACCGCCACCATGGCCATTAGCCAAGATGCCGTCATTCGGATGATTGACGAGCTTCTCCGTTTTGCTGGCGTTTCCGAAGATCCAGCAAAGAAACCAAGAAGTATGCTTGATGTTGGAAGTGGATTAGGTGGGACTTGTGTTTATGTAGCAAAGAAATATGATATACAATGTACAGGGATCACCATAAGCCCTAACCAAGTCAAATATGCTCAAGATTATGCAGCTACTGAAGGAGTAGAAAATAAGGTTTCTTTCGACTGTGGAGATGCCTTAGATATGCCTTATTCAGATGGTAAATTTGATGTGGTTTTCACCATTAATTGTATAAAACACGTTCATGACAAAGAAAAGTTCATTCGTGAGATGGTTCGGGTGGCAGCTCCCGGTGCTGCCATTATTATCGCGTCGCAAGCCCATCCAAATCTTTCTCCCGGGGAATCATTGAAACCACGGGACAAAAAAATACTGCAAAAAATATGTGACGGCGCCGGTGCTGTATCACTGTGTTCTTCAGATGATTATGTTAGGTGGTTAACTCCTCTTCCTGTTAAGGAGATCAAGGCCGCGGATTGGACTCAAAACATCACTCCACTTTATCCTCTATTGATGAAAGAAGCATTCACATGGAAGGGCTTCACGTCAATTGTGCTCAAGGGAGGATGGAGGGCTATCAACCTTATAAATGCAGTAAGGCTGGTGGCTAAGGCAGCAAATGACGGTATACTTAAGTTCGCTGTAGTTACGGGACGAAAATCAATA |
| *D4H* | Desacetoxyvindoline-4-hydroxylase | *C. roseus* | U71605.1 | ATGAAGGACTTGAACTTTCATGCTGCTACACTCTCAGAAGAAGAATCTTTAAGGGAATTGAAGGCTTTTGATGAGACAAAGGCTGGTGTAAAAGGGATTGTAGATACTGGGATAACCAAAATTCCACGTATCTTTATCGATCAACCAAAAAATCTTGACCGGATTTCAGTGTGTAGAGGAAAATCCGATATCAAGATTCCAGTTATAAACTTGAATGGCCTCAGTAGCAATTCAGAAATACGGCGTGAGATTGTGGAGAAAATTGGAGAAGCGAGTGAGAAATATGGATTCTTCCAGATAGTTAATCATGGGATTCCACAAGATGTTATGGATAAAATGGTAGATGGAGTTCGTAAGTTTCATGAACAAGATGATCAAATCAAGAGACAATATTACTCCCGTGACCGCTTCAACAAAAATTTTCTATATAGCAGTAATTACGTTTTGATTCCAGGAATTGCTTGCAATTGGAGGGATACTATGGAATGCATTATGAATTCTAATCAACCTGATCCTCAGGAATTCCCAGATGTATGCAGAGACATATTGATGAAGTACTCAAATTATGTAAGAAATTTGGGGCTTATTCTCTTTGAATTACTATCAGAAGCTTTGGGGCTCAAACCAAATCATCTTGAAGAAATGGATTGTGCTGAAGGACTTATACTTCTTGGTCATTACTACCCTGCATGCCCTCAACCAGAGTTGACATTTGGGACAAGCAAGCACTCAGACAGTGGTTTCTTAACCATACTTATGCAGGATCAAATTGGCGGCCTTCAAATTCTTCTTGAGAATCAATGGATTGATGTCCCCTTCATTCCTGGAGCTCTAGTTATTAACATTGCAGATCTTTTACAGCTGATCACGAACGACAAGTTCAAGAGTGTCGAACATAGAGTACTGGCAAACAAAGTTGGTCCGAGGATTTCTGTTGCTGTTGCTTTTGGTATAAAAACACAAACTCAAGAAGGGGTTTCGCCAAGATTGTACGGACCAATTAAAGAGTTAATATCAGAGGAAAATCCACCCATTTACAAGGAGGTCACTGTTAAAGATTTTATAACAATTCGATTTGCCAAACGCTTTGACGATAGCTCTTCCTTATCTCCTTTCAGGTTAAACAAT |
| *DAT* | Deacetylvindoline-4-O-acetyltransferase | *C. roseus* | AF053307.1 | ATGGAGTCAGGAAAAATATCGGTTGAGACAGAGACACTCTCCAAAACGTTGATCAAACCCTCTTCTCCAACCCCTCAATCCCTCAGCCGCTATAACCTCTCCTACAATGATCAAAACATATACCAAACGTGCGTATCCGTTGGTTTCTTTTACGAAAATCCCGACGGCATTGAAATTTCTACAATTCGTGAGCAGCTACAAAATTCGCTCTCAAAAACTCTTGTTTCTTATTATCCATTTGCTGGAAAAGTTGTTAAAAATGACTATATCCATTGCAATGACGATGGAATTGAGTTCGTGGAAGTTCGAATTCGTTGCCGCATGAATGATATTCTAAAGTATGAATTAAGATCATATGCCAGAGACCTAGTCCTTCCCAAACGGGTAACTGTTGGCAGTGAAGATACTACAGCTATTGTTCAACTAAGTCATTTTGATTGCGGAGGTTTGGCGGTTGCTTTTGGCATATCTCACAAAGTTGCTGATGGAGGTACAATTGCTTCGTTTATGAAGGATTGGGCTGCTTCTGCATGCTATTTATCTTCTTCTCATCACGTACCAACTCCGTTATTGGTTTCTGATTCCATATTTCCACGTCAAGATAATATAATTTGTGAACAATTTCCTACCTCCAAAAATTGTGTGGAAAAGACATTTATCTTCCCTCCGGAAGCCATAGAAAAGTTAAAATCTAAGGCCGTTGAGTTTGGTATTGAGAAACCTACTCGAGTAGAGGTTTTGACTGCTTTTCTCAGTCGATGTGCCACGGTAGCAGGGAAATCAGCAGCCAAGAATAATAATTGTGGCCAATCTTTGCCATTTCCAGTACTCCAAGCTATTAATTTACGTCCAATTTTGGAACTCCCACAAAATTCTGTGGGAAATTTGGTTTCAATTTATTTCTCACGTACAATTAAAGAAAATGATTATCTCAATGAAAAGGAATATACAAAACTAGTGATTAATGAGCTAAGGAAAGAAAAACAAAAGATAAAGAATCTTTCCCGAGAAAAGCTTACTTATGTAGCACAAATGGAAGAGTTCGTAAAAAGCTTGAAAGAGTTTGACATTAGCAACTTTTTGGATATCGATGCTTACCTTTCTGATAGTTGGTGTAGATTTCCCTTTTACGACGTTGATTTTGGATGGGGAAAGCCGATATGGGTATGCTTGTTTCAACCCTATATAAAAAATTGTGTTGTTATGATGGATTATCCATTTGGAGATGATTATGGAATCGAAGCAATAGTTTCCTTTGAACAAGAAAAGATGTCTGCCTTTGAGAAGAACGAACAGCTACTTCAATTTGTTTCTAATTAA |

**Supplementary References**

1 Liu, T. *et al.* Efficient production of vindoline from tabersonine by metabolically engineered *Saccharomyces cerevisiae*. *Commun. Biol.* **4**, 1089 (2021).

2 Liu, T. *et al.* Construction of ajmalicine and sanguinarine *de novo* biosynthetic pathways using stable integration sites in yeast. *Biotechnol. Bioeng.* **119**, 1314-1326 (2022).
